# Large-scale activation likelihood estimation meta-analysis of parkinsonian disorders

**DOI:** 10.1093/braincomms/fcad172

**Published:** 2023-05-30

**Authors:** Elizabeth G Ellis, Juho Joutsa, Jordan Morrison-Ham, Ellen F P Younger, Jacqueline B Saward, Karen Caeyenberghs, Daniel T Corp

**Affiliations:** Cognitive Neuroscience Unit, School of Psychology, Deakin University, Geelong, VIC 3220, Australia; Center for Brain Circuit Therapeutics, Department of Neurology, Psychiatry, and Radiology, Brigham and Women’s Hospital, Harvard Medical School, Boston, MA 02115, USA; Turku Brain and Mind Center, Clinical Neurosciences, University of Turku, Turku 20520, Finland; Turku PET Centre, Neurocenter, Turku University Hospital, Turku 20520, Finland; Cognitive Neuroscience Unit, School of Psychology, Deakin University, Geelong, VIC 3220, Australia; Cognitive Neuroscience Unit, School of Psychology, Deakin University, Geelong, VIC 3220, Australia; Cognitive Neuroscience Unit, School of Psychology, Deakin University, Geelong, VIC 3220, Australia; Cognitive Neuroscience Unit, School of Psychology, Deakin University, Geelong, VIC 3220, Australia; Cognitive Neuroscience Unit, School of Psychology, Deakin University, Geelong, VIC 3220, Australia; Center for Brain Circuit Therapeutics, Department of Neurology, Psychiatry, and Radiology, Brigham and Women’s Hospital, Harvard Medical School, Boston, MA 02115, USA

**Keywords:** Parkinson disease, atypical parkinsonian disorders, meta-analysis, activation likelihood estimation, neuroimaging

## Abstract

Parkinsonism is a feature of several neurodegenerative disorders, including Parkinson’s disease, progressive supranuclear palsy, corticobasal syndrome and multiple system atrophy. Neuroimaging studies have yielded insights into parkinsonian disorders; however, due to variability in results, the brain regions consistently implicated in these disorders remain to be characterized. The aim of this meta-analysis was to identify consistent brain abnormalities in individual parkinsonian disorders (Parkinson’s disease, progressive supranuclear palsy, corticobasal syndrome and multiple system atrophy) and to investigate any shared abnormalities across disorders. A total of 44 591 studies were systematically screened following searches of two databases. A series of whole-brain activation likelihood estimation meta-analyses were performed on 132 neuroimaging studies (69 Parkinson’s disease; 23 progressive supranuclear palsy; 17 corticobasal syndrome; and 23 multiple system atrophy) utilizing anatomical MRI, perfusion or metabolism PET and single-photon emission computed tomography. Meta-analyses were performed in each parkinsonian disorder within each imaging modality, as well as across all included disorders. Results in progressive supranuclear palsy and multiple system atrophy aligned with current imaging markers for diagnosis, encompassing the midbrain, and brainstem and putamen, respectively. PET imaging studies of patients with Parkinson’s disease most consistently reported abnormality of the middle temporal gyrus. No significant clusters were identified in corticobasal syndrome. When examining abnormalities shared across all four disorders, the caudate was consistently reported in MRI studies, whilst the thalamus, inferior frontal gyrus and middle temporal gyri were commonly implicated by PET. To our knowledge, this is the largest meta-analysis of neuroimaging studies in parkinsonian disorders and the first to characterize brain regions implicated across parkinsonian disorders.

## Teaser

Using a series of activation likelihood estimation meta-analyses in a large-scale data set of parkinsonian disorders (*n* = 132 studies), Ellis *et al*. localize robust brain abnormalities both distinct to and shared between individual parkinsonian disorders, across multiple imaging modalities.

## Graphical Abstract

**Graphical Abstract:**
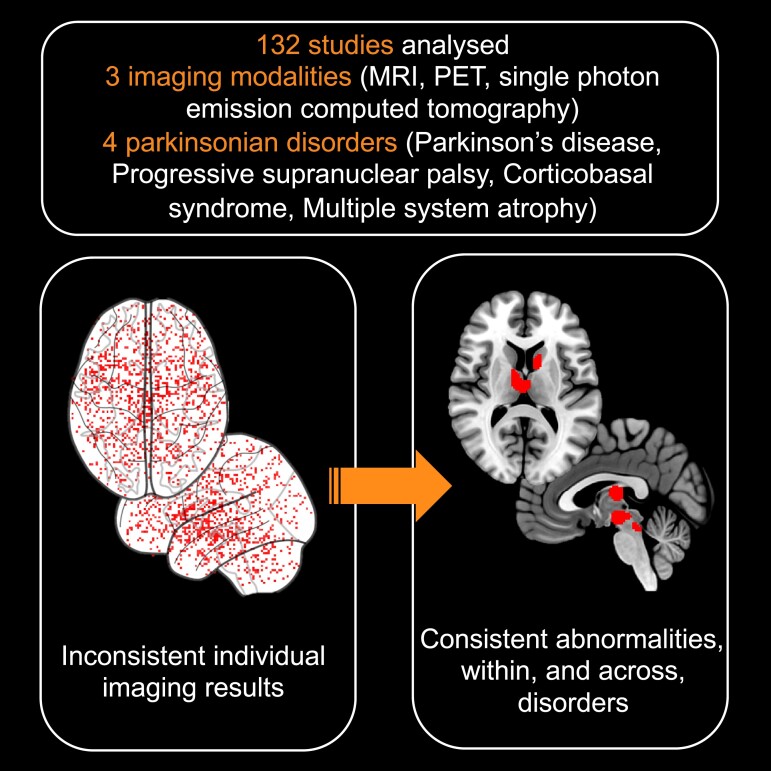

## Introduction

Parkinsonism is a debilitating neurological syndrome characterized by bradykinesia, tremor, rigidity and postural instability.^1^ The syndrome is most often caused by Parkinson’s disease—the second most prevalent neurodegenerative disease worldwide, with incidence rates predicted to double in the next 20 years.^2,3^ Parkinsonism can also be caused by several atypical parkinsonian disorders (APDs), including progressive supranuclear palsy (PSP), corticobasal syndrome (CBS) and multiple system atrophy (MSA).^2^ APDs are relatively rare and underdiagnosed, as the typical clinical characteristics can appear late in the course of the disease.^4,5^ Characterization of the commonly affected brain regions in these disorders is crucial to improving our understanding of their mechanisms.^4,6,7^

Traditionally, parkinsonism has been associated with dysfunction of the nigrostriatal tract.^7,8^ Consequently, treatments to alleviate parkinsonian symptoms act on components of this circuit, including dopaminergic medications and deep brain stimulation.^9,10^ Yet whilst these treatments have established efficacy in Parkinson’s disease, APDs patients show little to no response.^10,11^ There are some characteristic imaging findings, including the ‘hot cross bun’ sign of MSA, the ‘hummingbird’ sign of PSP and asymmetric cortical atrophy in CBS,^12–14^ but sensitivity of these findings especially in the early phases of the diseases is relatively weak and the characterization of affected brain regions in each condition remains incomplete. Clinical heterogeneity and methodological variability (e.g. imaging modalities and study settings) may contribute to heterogeneous neuroimaging findings.^6^ For example, brain alterations in Parkinson’s disease have been reported in all four lobes of the brain and often vary from study to study.^7^ Given that parkinsonism is a shared symptom complex, identifying converging brain abnormalities across parkinsonian disorders may clarify the common neural substrates.^15^

Meta-analytic methods have demonstrated the ability to collate heterogeneous imaging findings to converge upon a small number of key brain regions in neurological disorders.^16,17^ There is increasing evidence that symptoms share common neurobiological substrates^15,18^; however, this hypothesis has not yet been tested across parkinsonian disorders, with previous parkinsonian meta-analyses focusing on the individual disorders (e.g. Albrecht et al., Shao et al. and Yu et al.^19–21^). In addition, previous meta-analyses often only include studies of one neuroimaging modality (e.g. Albrecht et al., Shao et al. and Yu et al.^19–21^). Overall differences in search strategies, methods and variation in the sampled studies may limit the interpretability of findings between and across disorders.

Therefore, the aim of the present study was to analyse structural and molecular neuroimaging findings to characterize the brain regions affected in neurodegenerative parkinsonian disorders. We first performed meta-analyses to identify these regions within individual parkinsonian disorders across multiple imaging modalities and then performed a combined analysis across disorders to investigate possible shared neural substrates across parkinsonian disorders (Parkinson’s disease + PSP + CBS + MSA).

## Materials and methods

### Systematic search

**Figure 1:**
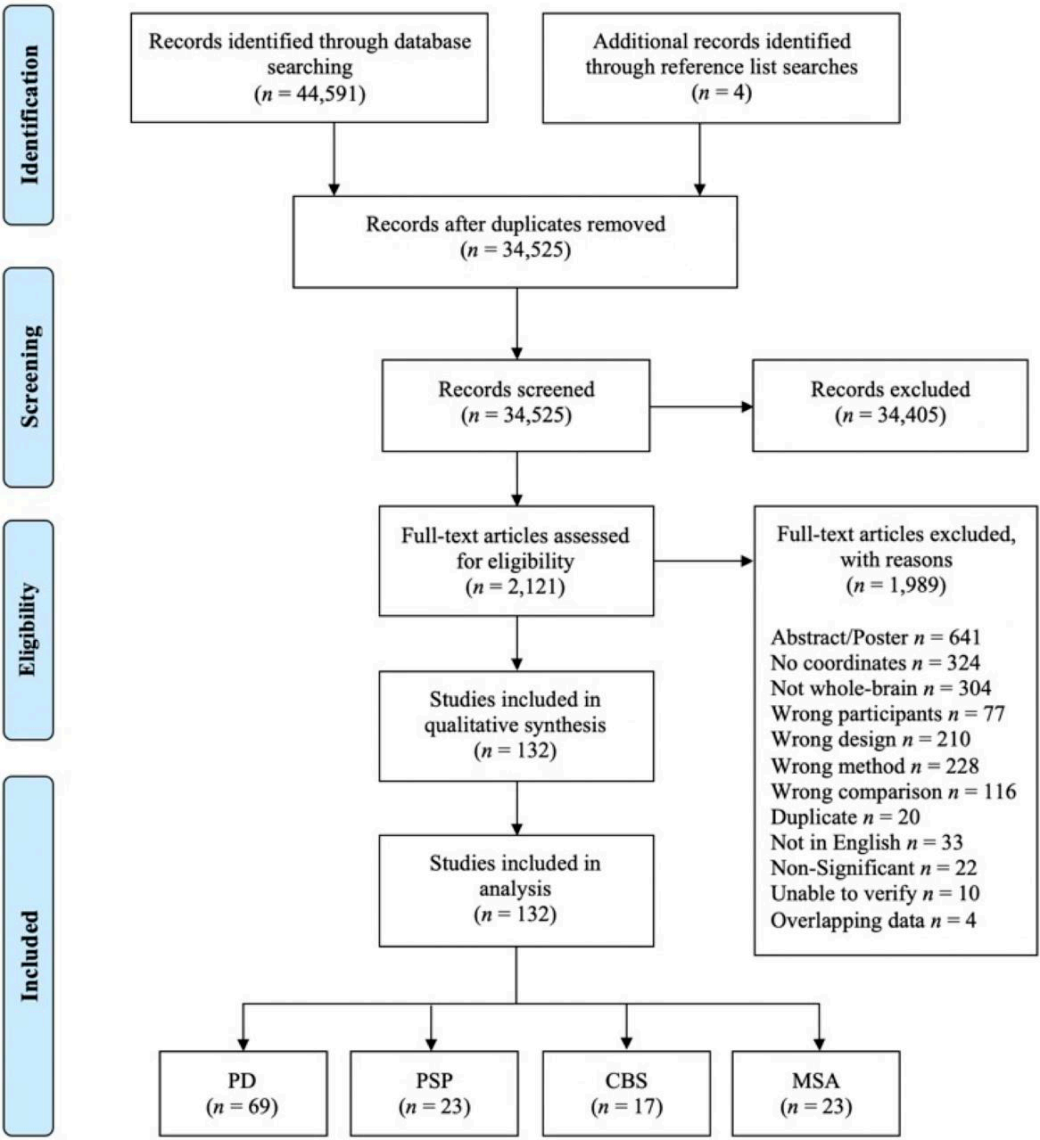
**PRISMA systematic literature search decision flowchart.** PRISMA systematic literature search decision flowchart displaying results of the four searches combined. Adapted from Moher *et al*.^22^ CBS = corticobasal syndrome; MSA = multiple system atrophy; PD = Parkinson’s disease; PSP = progressive supranuclear palsy.

Embase and MEDLINE Complete databases were searched for studies of neuroimaging in patients with idiopathic Parkinson’s disease, PSP, CBS and MSA. Four systematic searches were conducted (i.e. one for each disorder) following PRISMA guidelines^22^ (Fig. 1 and Supplementary Figs. 1–4). No language or year limiters were applied. Studies were identified using a combination of keywords relating to the diagnosis of focus, neuroimaging techniques and imaging outcomes, for example, ‘parkinson* disease’, ‘magnetic resonance imaging’, ‘single-photon emission computed tomography’ and ‘atrophy’ (for full syntax, see Supplementary Table 1). Studies were screened at title/abstract level using EndNote (Version X9) and Rayyan^23^ software, and full-text articles were screened in EndNote. Reference lists of all eligible studies were assessed for studies missed by the initial search.

#### Inclusion and exclusion criteria

To ensure objectivity in brain regions identified, studies must have (i) reported whole-brain neuroimaging analysis;^17^ (ii) reported anatomical coordinates of significant brain abnormalities in stereotactic space; (iii) provided information on how the clinical diagnosis was made; and (iv) employed a healthy participant comparison group.

Exclusion criteria were (i) studies using neurotransmitter or protein ligands and neuromelanin-sensitive MRI, excluded from the current analysis to avoid biased identification of regions dense in certain neurotransmitters, proteins and melanin, respectively; (ii) studies examining parkinsonism resulting from confirmed pathology (e.g. drug induced, familial or a consequence of other disease); (iii) studies not in English; and (iv) case studies, reviews and meta-analyses.

### Statistical analysis

ALE meta-analyses were conducted as per the revised algorithm presented by Eickhoff *et al*. (2012)^16^, implementing a random-effects model,^17^ refined permutation testing and corrections for multiple comparisons. Prior to ALE analyses, Talairach coordinates were transformed into MNI space using the Yale BioImage Suite converter^24^ (https://bioimagesuiteweb.github.io/webapp/mni2tal.html). Analyses were performed using GingerALE (v3.2; http://www.brainmap.org/ale/). In this revised algorithm, each coordinate (‘foci’) is represented by a Gaussian probability distribution reflecting the underlying spatial uncertainty of each point. The width of each distribution is determined by the sample size of the study from which they were extracted.^16^ Voxel-wise pooling of these probability distributions occurs to generate whole-brain ‘modelled activation’ (MA) maps—within which, each voxel represents the probability that a coordinate exists at that location (e.g. the probability that volume loss occurred at that given voxel). The union of these individual MA maps was computed and referred to as the ‘ALE map’. To determine significance, the ALE map was then tested against the null distribution of 1000 simulated data sets with identical characteristics to the input (i.e. number of studies, sample sizes and number of foci) but with randomly distributed foci.^17^ Within a cluster-level design, MA values are distributed throughout the brain to determine which clusters of convergence from the included studies are significantly larger in their spatial extent than could be expected by chance.

Each analysis was performed with cluster-forming thresholds of *P* < 0.001 (uncorrected), 1000 permutations and a cluster-level inference threshold correcting for multiple comparisons with family-wise error (FWE) rate at *P* < 0.05.^16^ Thresholds are in accordance with recommendations regarding both the sensitivity and specificity of cluster-level inference and corrections for multiple comparisons.^25^

Recent evidence suggests that PET imaging may be more sensitive to brain alterations in patients with Parkinson’s disease compared with structural MRI.^6^ However, structural MRI has been far more commonly used to investigate brain abnormalities in parkinsonian disorders. Therefore, to provide a comprehensive overview of brain regions implicated in these disorders, we included coordinates from PET, MRI and single-photon emission computed tomography (SPECT) studies, as per previous meta-analyses.^18,26^

Meta-analyses were performed using two contrasts: first, coordinates of *decreased* volume, metabolism or perfusion in patients compared with healthy controls (HC) (i.e. parkinsonian disorders < HC; Parkinson’s disease < HC; PSP < HC; CBS < HC; and MSA < HC); second, using coordinates of *increased* volume, metabolism or perfusion in each patient group compared to HC. Cluster regional labels for all analyses were defined using Harvard–Oxford cortical and subcortical structural atlases (FSL: https://fsl.fmrib.ox.ac.uk/fsl/fslwiki/Atlases). Some contrasts comprised a small number of studies for meta-analysis (e.g. only two studies using PET imaging in CBS patients satisfied inclusion criteria). Given that unreliable estimates may result from a low *n* in ALE meta-analysis,^25,27^ we set a cut-off of 10 studies in order to conduct ALE meta-analysis. Contrasts with fewer than 10 studies were included for qualitative review only. This cut-off was determined to balance our intent to analyse as many contrasts as possible, taking into account previous ALE meta-analyses with smaller sample sizes (six to nine studies^20,21,28^), and the increased risk of Type I error associated with an underpowered sample.^25^

## Results

### Study selection

A total of 44595 articles were assessed for eligibility (Fig. 1). After duplicate removal, 34 525 articles were screened at title and abstract level, 2121 at full-text, with 132 studies meeting inclusion criteria (69 Parkinson’s disease; 23 PSP; 17 CBS; and 23 MSA). PRISMA systematic search flowcharts for each search (per disorder) are provided in Supplementary Figs. 1–4. The final search was conducted on 22 June 2022.

The 132 included studies comprised 7545 participants and 2190 coordinates of significant differences between patients and controls (Table 1). Imaging modalities of the included studies were whole-brain structural MRI (*n* = 85), perfusion and metabolism PET (*n* = 32) and SPECT (*n* = 15). Characteristics of all included studies, including neuroimaging modality, participants and diagnostic criteria, are provided in Supplementary Table 2.

**Table 1:** Cohorts included in review

Cohort	Studies	Participants		Total coordinates
		patients	Controls	
Parkinsonian disorders	132	4211	3334	2190
PD	69	2817	1886	963
PSP	23	363	425	355
CBS	17	324	396	210
MSA	23	707	627	450

*‘*Parkinsonian disorders’ encompasses all four parkinsonian disorders. CBS = corticobasal syndrome; MSA = multiple system atrophy; PD = Parkinson’s disease; PSP = progressive supranuclear palsy.

### Qualitative analysis

Overall, 12 contrasts did not comprise enough studies for meta-analysis and were included for qualitative review only (Table 2; italicized authors in Supplementary Table 2). Studies included for qualitative review reported 939 coordinates of significant brain abnormality between patients and HC. All PET imaging studies assessed alterations in glucose metabolism, and all SPECT studies examined differences in perfusion. Populations sampled by these studies included patients with various comorbidities, such as pathological gambling,^29^ cognitive impairment^35^ and parkinsonian and cerebellar subtypes of MSA.^43,48,51,56,62^ Regions reported to be abnormal between patients and controls within each contrast were distributed throughout the brain; commonly reported regions are noted in Table 2. All studies were meta-analysed in the ‘all parkinsonian disorders’ combined meta-analyses.

**Table 2:** Contrasts qualitatively reviewed

Contrast	*n*	Main Findings
**PD < HC** SPECT	8	Cortical alterations in frontal (6/8 studies),^29–34^ parietal (6/8),^29,32–36^ occipital (6/8)^29,30,32,33,35,36^ and temporal lobes (5/8)^30,32,34,37,38^
**PD > HC** SPECT	5	Basal ganglia, cerebellum^29,32,33,37,38^
**PD > HC** MRI	5	Cerebellum (4/5 studies)^39–42^
**PSP </> HC** SPECT	2	No commonalities identified^43,44^
**PSP < HC** PET	6	Midbrain, frontal lobes^45–50^
**PSP > HC** PET	2	Cerebellum^48,49^
**MSA < HC** PET	6	Putamen, cerebellum (5/6 studies)^48,51–54^
**MSA > HC** PET	2	No commonalities identified^48,51^
**MSA < HC** SPECT	3	Putamen (2/3 studies)^34,55^
**MSA > HC** MRI	2	No commonalities identified^56,57^
**CBS < HC** PET	4	Motor cortices (4/4)^48,50,58,59^
**CBS < HC** SPECT	2	Superior frontal gyrus^60,61^

All studies qualitatively reviewed are noted in italics in the study characteristics table (Supplementary Table 2). CBS = corticobasal syndrome; HC = healthy controls; MSA = multiple system atrophy; PD = Parkinson’s disease; PSP = Progressive supranuclear palsy; SPECT = single-photon emission computed tomography.

### Activation likelihood estimation meta-analyses

Five contrasts met our sample size threshold for ALE meta-analysis (≥10 studies). Analyses were conducted across studies reporting the results of MRI in patients with Parkinson’s disease, PSP, CBS and MSA compared with controls; in addition, one PET meta-analysis was conducted on studies of patients with Parkinson’s disease. Significant findings are detailed in Table 3; for a list of all studies contributing to each significant cluster, see Supplementary Table 3.

**Table 3:** Significant ALE meta-analysis findings in parkinsonian disorder*s*

Contrast	Region		*x*	*y*	*z*	Volume (mm^3^)	ALE value	Convergence *n* (%)
**PET** **PD < HC**	1	L. Mid. Temporal G.	−44.7	−64.4	35.8	2088	0.0251	8 (47%)
Number of experiments in analysis = 17	2	L. Caudate	−14.3	13.1	6.6	1000	0.0251	5 (29.4%)
	3	R. Inferior Frontal G.	57.4	16.4	23.6	680	0.0253	3(17.6%)
	4	R. Middle Frontal G.	34	22	42.8	664	0.0223	3(17.6%)
**MRI** **PSP < HC**	1	Bilat. Thalamus/Red nucleus	2.2	−14.1	−0.9	11 072	0.0488	14 (87.5%)
Number of experiments in analysis = 16	2	R. Insula	43.5	17.2	4.1	1040	0.023	4 (25%)
	3	L. Caudate	−10.7	6.2	12.9	952	0.0257	6 (37.5%)
	4	L. Brainstem	−7.4	−34.9	−13.5	816	0.0231	4 (25%)
	5	L. Insula	−37	16.9	3.5	808	0.0218	4 (25%)
**MRI** **MSA < HC**	1	Brainstem	1.2	−33.8	−18.9	896	0.0256	4 (30.8%)
Number of experiments in analysis = 13	2	L. Putamen	−22.7	11.6	−6.4	776	0.0209	4 (30.8%)
	3	Brainstem	−12	−35.3	−32	640	0.0162	5 (38.5%)
**MRI** **Parkinsonian disorders < HC**	1	R. Thalamus^*4/4^	5.4	−11.9	13.5	2744	0.0498	12 (14.3%)
Number of experiments in analysis = 84	2	L. Caudate^*3/4^	−26.3	13.9	6.3	2312	0.0385	16 (19%)
	3	Bilat. Midbrain/Red nucleus^*2/4^	1.6	−17.5	−9	1432	0.0538	12 (14.3%)
	4	L. Amygdala^*3/4^	−19.7	−8.4	−11.7	1016	0.0305	10 (11.9%)
	5	R. Parahippocampal G., Amygdala^*3/4^	19	−12.4	−14.4	1008	0.0349	7 (8.3%)
	6	L. Brainstem^*2/4^	−3.2	−33.4	−16	856	0.0302	6 (7.1%)
**PET** **Parkinsonian disorders < HC**	1	L. Lateral Occipital cortex^*3/4^	−45.5	−64.2	34.3	2344	0.0319	10 (30.3%)
Number of experiments in analysis = 33	2	L. Caudate^*4/4^	−14.9	11.9	7.5	2056	0.0414	11 (33.3%)
	3	R. Caudate^*4/4^	16.2	13.7	4.9	1840	0.0345	10 (30.3%)
	4	R. Inferior Frontal G.^*4/4^	57.1	15	23.4	1296	0.0403	8 (24.2%)
	5	R. Middle Frontal G.^*2/4^	34.1	20.3	46	848	0.0228	5 (15.2%)
	6	R. Lateral Occipital cortex^*3/4^	42	−60	47.3	752	0.0212	4 (12.1%)
**Parkinsonian disorders>HC**	1	R. Middle Temporal G.^*4/4^	39.5	−31.8	−2.8	1200	0.0159	5 (45.5%)
Number of experiments in analysis = 11	2	L. Insular cortex^*3/4^	−32.6	−18.5	21.7	792	0.015	4 (36.4%)
	3	L. Inferior Occipital G.^*3/4^	−40.3	−81.6	1.6	648	0.0178	3 (27.3%)

All clusters reported were significant at *P* < 0.05 family-wise error corrected. Centre of gravity provided in MNI *x*, *y* and *z* coordinates. Some studies included multiple patient groups and therefore performed multiple experiments. Convergence *n* = number of experiments contributing to clusters. Bilat. = bilateral; G. = gyrus; HC = healthy controls; Inf. = inferior; L. = left; Mid. = middle; MNI = Montreal Neurological Institute; MSA = multiple system atrophy; PD = Parkinson’s disease; PSP = progressive supranuclear palsy; R. = right. * denotes the number of disorders in the parkinsonian analyses that contributed to the significant result. For a list of studies contributing to each cluster, see Supplementary Table 3.

#### Parkinson’s disease ALE meta-analysis

##### MRI

The Parkinson’s disease MRI < HC meta-analysis was non-significant (*P* > 0.05 FWE corrected). The Parkinson’s disease MRI > HC meta-analysis was also non-significant.

##### PET

**Figure 2:**
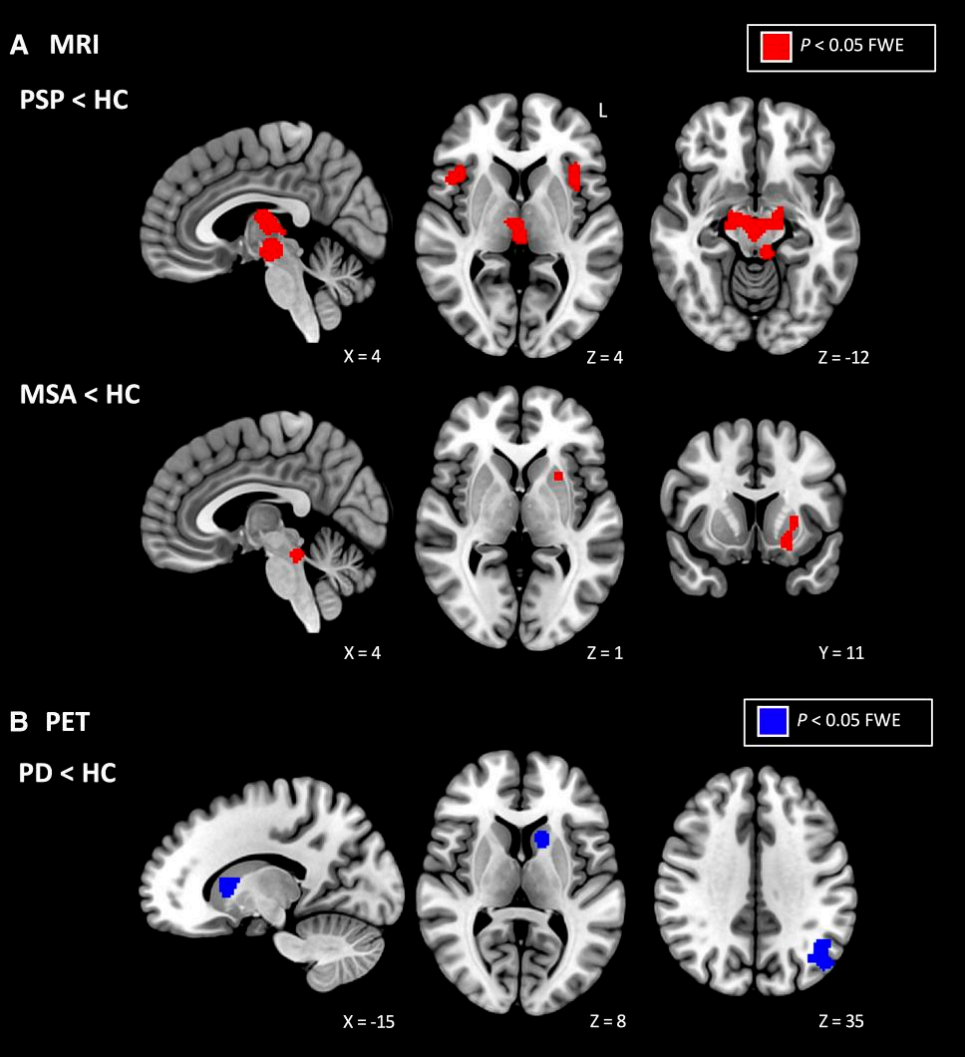
**Meta-analysis results in individual parkinsonian disorders**. (**A**) Meta-analyses of MRI imaging, patients < HC. Alteration of the red nucleus was characteristic of PSP patients, whilst the brainstem and putamen were the most commonly implicated regions in patients with MSA. (**B**) Meta-analysis of PET imaging in Parkinson’s disease; patients < HC. The middle temporal gyrus and left caudate were consistently implicated by PET imaging in Parkinson’s disease patients. All significant clusters are reported in Table 3. FWE = family-wise error; L = left; MSA = multiple system atrophy; PD = Parkinson’s disease; PSP = progressive supranuclear palsy.

The Parkinson’s disease < HC meta-analysis of PET studies identified four significant clusters within the left middle temporal gyrus, caudate and right inferior frontal and middle frontal gyri (*P*< 0.05 FWE corrected; Table 3 and Fig. 2B).

##### SPECT

No meta-analyses were performed of SPECT studies in Parkinson’s disease due to insufficient sample sizes (Parkinson’s disease < HC *n* = 8 and Parkinson’s disease > HC *n* = 5).

#### PSP ALE meta-analysis

##### MRI

Five significant clusters were identified for the PSP < HC contrast, encompassing bilateral thalami, right insula, left caudate, left anterior cerebellar lobe and left insula/claustrum (*P* < 0.05 FWE corrected; Fig. 2A, first row, and Table 3). No analysis was conducted for the opposite contrast as no studies reported coordinates of increased grey/white mater volume.

##### PET and SPECT

No meta-analyses were performed on PET (*n* = 6) or SPECT (*n* = 2) studies in PSP due to insufficient sample sizes.

#### CBS ALE meta-analysis

##### MRI

The meta-analysis of MRI studies in the CBS < HC contrast was non-significant. As no studies reported ‘increases’ in grey/white matter volume, no meta-analysis was conducted.

##### PET and SPECT

No meta-analyses were performed on PET (*n* = 5) or SPECT (*n* = 2) studies in CBS due to insufficient sample sizes.

#### MSA ALE meta-analysis

##### MRI

Three clusters were significant for the MSA < HC MRI meta-analysis, two of which were located within the brainstem and one cluster within the left putamen (*P* < 0.05 FWE corrected) (Fig. 2A, second row, and Table 3). No meta-analysis was performed for the MSA > HC contrast as only two studies reported coordinates for that contrast.

##### PET and SPECT

No meta-analyses were performed on PET (MSA < HC *n* = 6 and MSA > HC *n* = 3) or SPECT (*n* = 3) studies in MSA due to insufficient sample sizes.

#### ALE meta-analysis of parkinsonian disorders

Finally, meta-analysis was performed across all 132 included studies of parkinsonian disorders within each neuroimaging modality.

##### MRI

**Figure 3:**
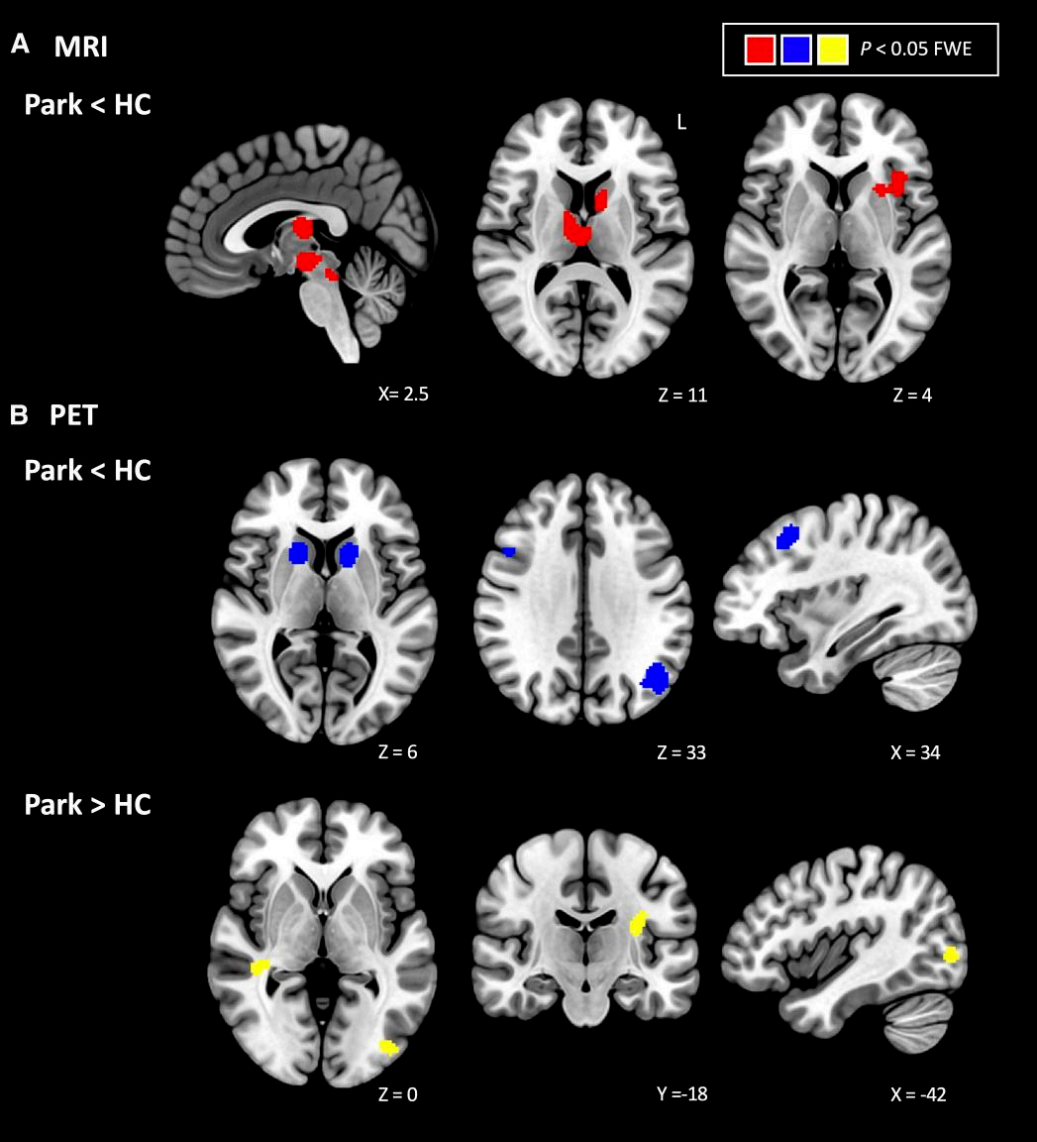
**Meta-analysis findings across parkinsonian disorders**. (**A**) Meta-analysis of MRI imaging in all parkinsonian disorders, patients < HC (top row). (**B**) Meta-analyses of PET imaging in all parkinsonian disorders, patients < HC (middle row) and patients > HC (bottom row). FWE = family-wise error; HC = healthy controls; Park = parkinsonian disorders.

Six significant clusters were identified in the parkinsonian disorders < HC analysis of MRI studies (*P* < 0.05 FWE corrected) (Fig. 3 and Table 3), found throughout the thalamus, basal ganglia, parahippocampal gyrus, brainstem and midbrain. The opposite contrast, examining increases in patients compared to controls, was non-significant.

##### PET

Six clusters were identified for the patients < HC contrast (*P* < 0.05 FWE corrected) (Fig. 3B and Table 3), found throughout the caudate, lateral occipital cortices and inferior and middle frontal gyri. Three clusters were significant in the opposite contrast (patients > HC) (*P* < 0.05 FWE corrected) (Fig. 3C and Table 3), including the middle temporal gyrus, insular cortex and inferior occipital gyrus.

##### SPECT

The Parkinsonian disorders < HC analysis of SPECT studies was non-significant. Only six studies reported increases in patients compared with controls using SPECT imaging, and therefore no meta-analysis was conducted on this contrast (patients > HC).

Overall, the combined parkinsonian disorder meta-analysis revealed several regions that were reported across all four parkinsonian disorder cohorts. Specifically, abnormality of the thalamus was implicated in MRI studies of all disorders, whilst alteration to bilateral caudate, inferior frontal and middle temporal gyri was implicated by PET studies in all disorders. The caudate was the only significant cluster identified by the combined parkinsonian disorder meta-analyses, which was consistent across multiple modalities (MRI and PET).

## Discussion

The present study conducted a series of whole-brain ALE meta-analyses to identify consistent regions of brain abnormality within, and across, parkinsonian disorders. Results in PSP and MSA aligned with current clinical imaging markers. PET studies of Parkinson’s disease patients most consistently reported abnormality of the middle temporal gyrus. Several regions were found to be common to all disorders by our analysis of all parkinsonian disorders combined, specifically abnormality of the thalamus reported by MRI studies, and bilateral caudate, inferior frontal and middle temporal gyri alterations implicated by PET. Our findings highlight individual patterns of brain abnormality within each disorder, whilst also demonstrating that a small number of brain regions are implicated across all of the included parkinsonian disorders.

### Parkinson’s disease

Meta-analysis of PET imaging studies in Parkinson’s disease revealed hypometabolism of the left middle temporal gyrus, caudate and right inferior and middle frontal gyri to be the most robust regions of abnormality, consistent with prior meta-analysis.^6^ In contrast to previous meta-analyses,^6,20,21,28^ our MRI meta-analysis did not identify any significant clusters. These findings are in alignment with the conclusion of Albrecht *et al.*^6^ suggesting that PET imaging is more sensitive to brain abnormalities in Parkinson’s disease patients than MRI.

### Progressive supranuclear palsy

PSP was characterized by midbrain abnormality, consistently reported by MRI studies, aligning with previous meta-analyses.^20,21^ Midbrain atrophy is a hallmark PSP pathology,^2,63,64^ with imaging markers such as a reduced midbrain-to-pons ratio and the hummingbird sign, used as tools for differential diagnosis.^65,66^ In addition, structural and functional imaging studies have previously implicated abnormality of the caudate and thalamus in postural instability and ocular motor dysfunction, domains commonly affected in PSP.^67,68^

### Corticobasal syndrome

Whilst our meta-analysis of MRI studies in CBS was non-significant, previous meta-analyses have reported more distributed alterations in CBS, involving the thalamus, insula and multiple cortical sites.^19,21^ It is possible that our stricter threshold resulted in non-significant findings, given that previous meta-analyses applied less stringent statistical thresholds.^25^ There were insufficient PET and SPECT studies of CBS to conduct independent meta-analyses for those modalities; however, general trends were present across the included studies; consistently describing the hallmark asymmetrical abnormalities^48,58,69,70^ and consistent implication of the motor cortices by PET imaging.^48,50,58,59^

### Multiple system atrophy

MSA was characterized by consistent abnormality within the brainstem and putamen, in agreement with previous meta-analyses.^21,28,71^ Identification of robust abnormality of the putamen and brainstem aligns with imaging markers used for differential diagnosis, including the distinctive MSA ‘hot cross bun’ sign within the pons.^12^ Of further interest, the included imaging studies of MSA examined samples of patients with both the parkinsonian (*n* = 9) and cerebellar (*n* = 6) MSA subtypes (see Supplementary Table 2 for study characteristics). Whilst samples were too small for independent meta-analysis, all six studies examining MSA-cerebellar patients, reported abnormality of the cerebellum^56,62,72–75^ and across the nine studies with MSA-parkinsonian patients, alterations to the cerebellum,^74–78,51^ and basal ganglia,^75–79,51^ were consistently reported.

### Common findings across parkinsonian disorders

Abnormality of the thalamus was reported in MRI studies of all four parkinsonian disorders whilst PET imaging consistently implicated bilateral caudate, right inferior frontal and middle temporal gyri. Identification of these shared regions may support the notion that parkinsonian symptoms share common neural substrates, which could reflect their involvement in the overlapping motor and non-motor symptoms amongst parkinsonian disorders. For example, the thalamus has an established role in connecting and modulating basal ganglia circuitry and is associated with a range of functions, including motor control and behaviour modulation.^80^ In addition, the caudate has extensive sensorimotor connections yet is also involved in cognitive and associative functions.^81^ Both of these subcortical regions have been localized in previous meta-analyses of CBS, PSP and Parkinson’s disease,^6,19,20^ and as sites of lesions causing parkinsonism.^82–85^ Further, although the inferior frontal and middle temporal gyri may not have established roles in the classical parkinsonian syndrome, both regions have been implicated in studies of individual disorders (e.g. in Parkinson’s disease, PSP and MSA^48,86,87^). These cortical regions have also been linked to behavioural inhibition^88^ and freezing of gait,^89^ respectively.

Although these shared brain regions could suggest that these regions play a role in parkinsonian symptoms, it is also possible that these sites may be common across parkinsonian disorders because of particular vulnerability to degeneration, as opposed to them having a direct involvement in symptom generation.^19,90^ Though this must be acknowledged, our hypothesis that these common regions could be involved in the shared substrate may be supported by their previous implication in lesion-induced parkinsonism (e.g. lesions to the thalamus^82^ and caudate^83,84^) and associations with motor deficits in functional studies (e.g. the middle temporal gyrus and gait^89^).

### Differences in localizations between neuroimaging modalities

Meta-analyses within each imaging modality of the same disorder localized different brain regions (e.g. an MRI meta-analysis in parkinsonian disorders revealed clusters within largely subcortical regions whilst the PET meta-analysis identified clusters across cortical and subcortical regions). Given that each modality examines different neural mechanisms (i.e. alterations to grey/white matter volume with MRI and cerebral metabolism or perfusion with PET and SPECT),^91^ differences in the brain structures they implicate are not unexpected. The caudate was the only region that was identified across meta-analyses of multiple imaging modalities (MRI and PET). Despite both modalities implicating subcortical and cortical alterations, the clusters identified were not within the same brain structures. One explanation of these differences is that PET imaging may be more sensitive to brain alterations than MRI in Parkinson’s disease.^6^

### Localization of brain networks versus brain regions

Whilst several of our meta-analyses demonstrated high convergence, there were still numerous studies that did not contribute to the localized clusters. For example, only 8 of 17 (47%, Table 2) PET studies in Parkinson’s disease contributed to the significant cluster in the middle temporal gyrus. Although these findings may suggest a lack of reproducibility,^92^ it is also possible that the remainder of these studies’ coordinates are still functionally connected to the clusters that we identified.^18^ Recent studies have shown that complex symptoms that failed to localize to discrete brain regions successfully localize to functionally connected brain networks.^18,93,94^ It is possible that such network mapping analyses may be useful in reconciling apparently inconsistent findings of neuroimaging studies in parkinsonian disorders.

### Limitations

Our meta-analysis has a number of limitations that should be discussed. First, most of the included studies did not have diagnoses confirmed by autopsy. Due to the poor sensitivity of clinical APDs diagnoses and overdiagnosis of Parkinson’s disease,^4^ misdiagnoses within the published studies are possible. Second, ALE results are driven by coordinates of statistically significant differences between patients and controls. Whether patient differences reach significance in each study will have been influenced by methods decided by these studies’ authors (e.g. image processing, statistical thresholds applied), which were not uniform across the included studies. Whilst the ALE technique partially controls for variance in study methodologies, it does not do so completely. Similarly, the method does not account for the possible contribution of confounding variables (e.g. age/sex) as one could in traditional meta-analysis. Future coordinate-based meta-analytic techniques should aim to incorporate control of covariates. Third, without the use of neurodegenerative control conditions, it is possible that some of the regions identified by our parkinsonian analyses may be commonly abnormal across disorders because of vulnerability of certain regions to degeneration rather than due to their specific involvement in the generation of shared symptoms.^1,95^

## Conclusion

To our knowledge, this is the largest whole-brain ALE meta-analysis of parkinsonian disorders and the first to characterize brain regions implicated across disorders, and within multiple neuroimaging modalities. Localizations aligned with current diagnostic imaging markers, isolating the midbrain in PSP, and the brainstem in MSA. PET studies most consistently reported alteration to the middle temporal gyrus in Parkinson’s disease patients. Importantly, we also demonstrated several regions that were abnormal across these parkinsonian disorders, including the thalamus, caudate, inferior frontal and middle temporal gyri. These findings help to illuminate the brain regions that are abnormal in parkinsonian disorders and highlight a set of common brain regions across disorders that may contribute to shared parkinsonian symptoms.

## Supplementary Material

fcad172_Supplementary_Data

## Data Availability

The data that support the findings of this study are available from the corresponding author upon request. Code used via Ginger ALE version 3.2 is available through http://brainmap.org.

## Abbreviations

ALE =: activation likelihood estimation
APDs =: atypical parkinsonian disorders
CBS =: corticobasal syndrome
FWE =: family-wise error rate
HC =: healthy controls
MA =: modelled activation
MSA =: multiple system atrophy
PSP =: progressive supranuclear palsy
SPECT =: single-photon emission computed tomography
