# Supplementary material for: Large-scale activation likelihood estimation meta-analysis of parkinsonian disorders
Source: Brain Commun. 2023 May 30;5(3):fcad172. doi: 10.1093/braincomms/fcad172 (PMC10265724; doi:10.1093/braincomms/fcad172)
Supplement: fcad172_Supplementary_Data [file fcad172_supplementary_data.pdf]

Supplementary Material

Supplementary Figure 1

PRISMA Systematic Literature Search Decision Flowchart for Parkinson's Disease.

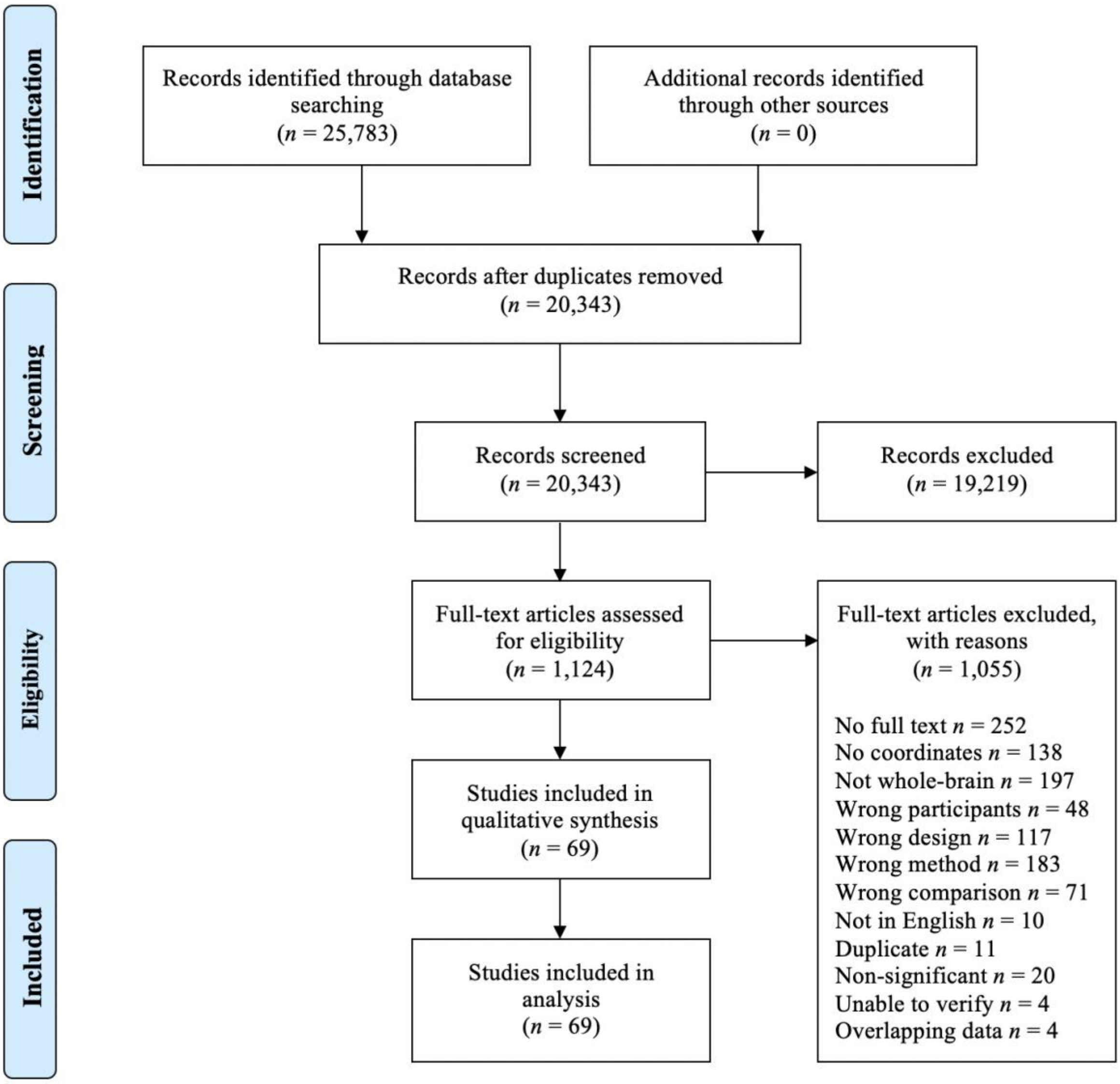

Note. The reason 'Wrong method' combines: *wrong imaging technique* (113) and *wrong analysis* (15). 'Duplicate' combines: *duplicate records* (6), *duplicates of the original search found in the 2022 updated search (3)* and *duplicated samples* (4) published papers using the same participant cohorts, where possible the largest appropriate sample has been chosen.

Supplementary Figure 2

PRISMA Systematic Literature Search Flowchart for Progressive Supranuclear Palsy

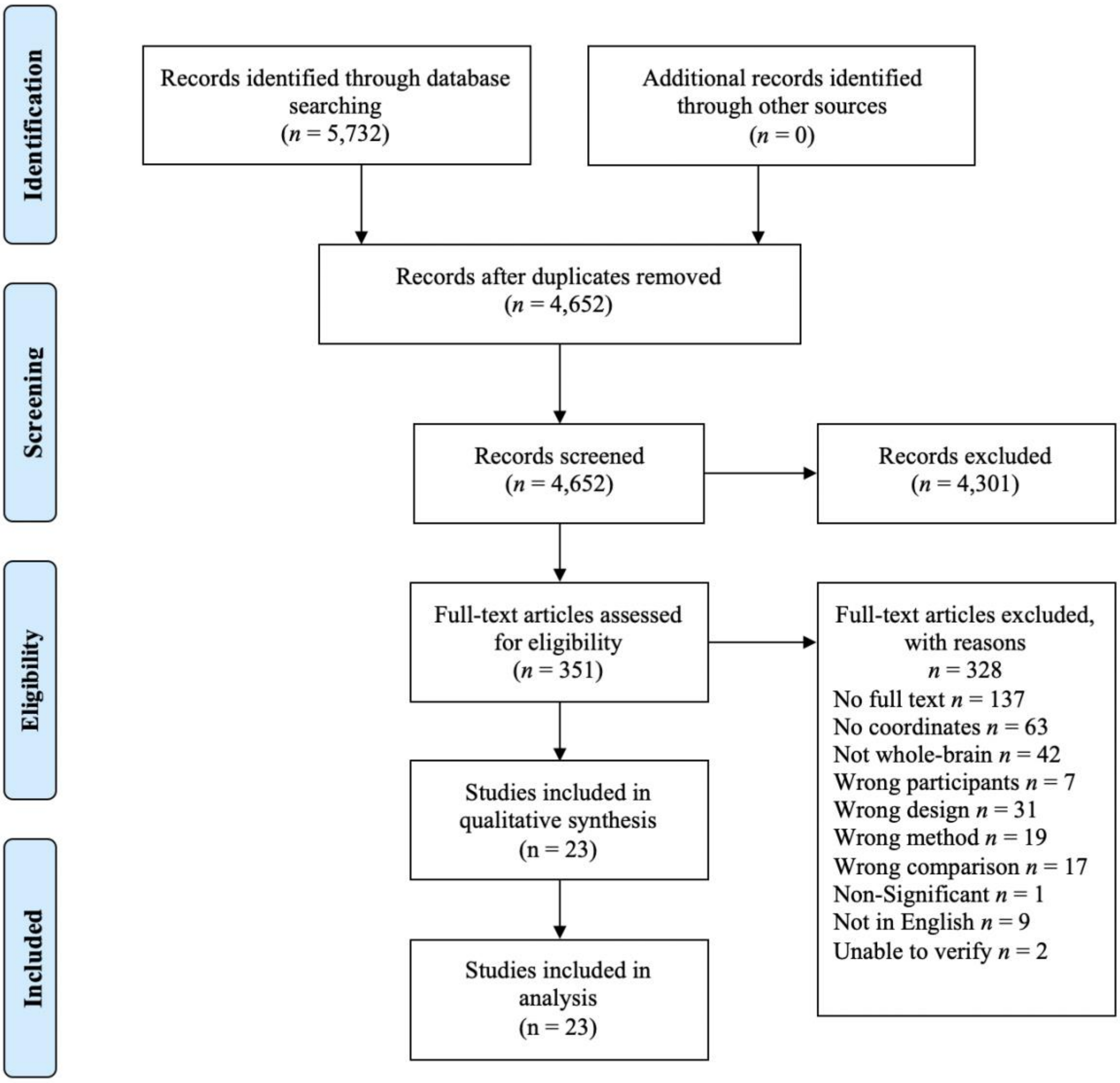

Note. The reason ‘Wrong method’ combines: *Wrong imaging technique* (11); *Results correlated with a task/symptom/effect* (1) and *Wrong analysis* (5).

Supplementary Figure 3

PRISMA Systematic Literature Search Flowchart for Corticobasal Syndrome

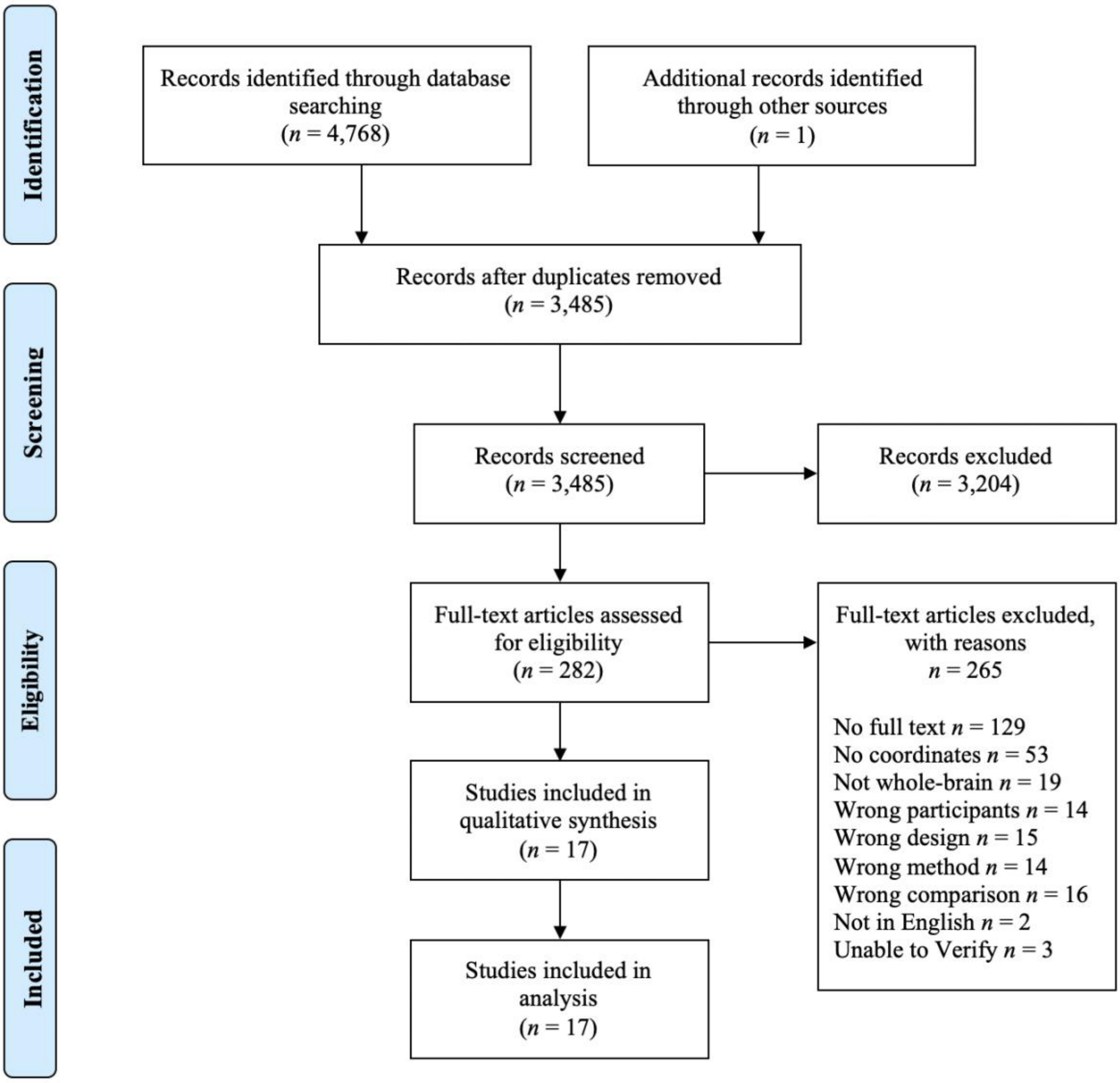

Note. The reason 'Wrong method' combines: *Wrong imaging technique* (7); *Results correlated with a task/symptom/effect* (7).

Supplementary Figure 4

PRISMA Systematic Literature Search Flowchart for Multiple System Atrophy

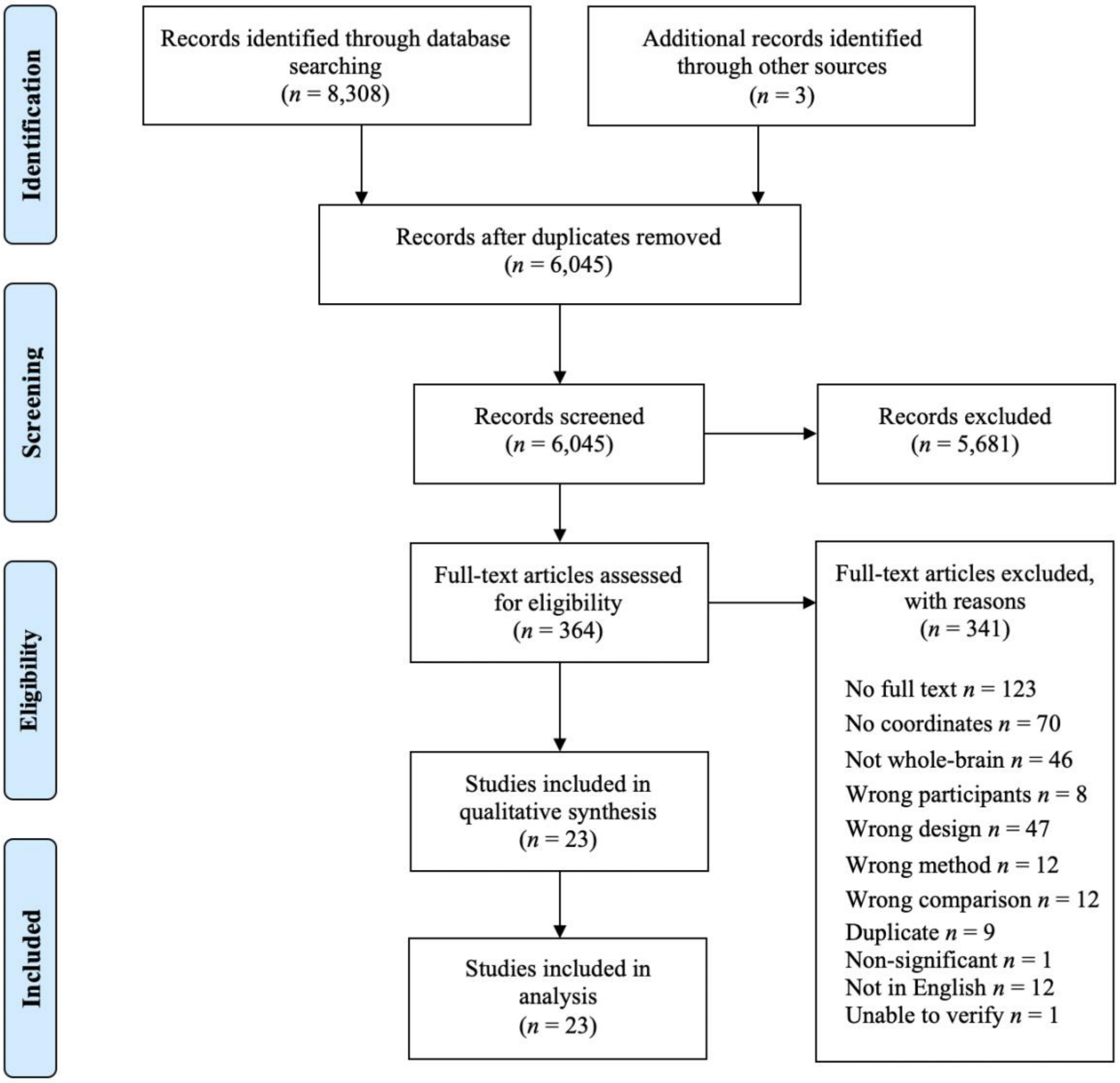

Note. The reason 'Duplicate' combines: *duplicate records* (4) and *duplicate samples* (2) papers that have published using the same participant cohorts, where possible the largest appropriate sample has been chosen.

Supplementary Table 1 *Systematic Search Syntax for Individual Parkinsonian Disorders*

Database	Syntax
Embase	#1 AND #2 AND #3
#1	('mri':ti OR 'magnetic resonance imaging':ti OR 'vbm':ti OR 'voxel based morpho*':ti OR 'spect':ti OR 'single photon emission computed tomography':ti OR 'positron emission tomography':ti OR 'pet':ti OR 'hypometabolism':ti OR 'hypoperfusion':ti OR 'mri':ab OR 'magnetic resonance imaging':ab OR 'vbm':ab OR 'voxel based morpho*':ab OR 'spect':ab OR 'single photon emission computed tomography':ab OR 'positron emission tomography':ab OR 'pet':ab OR 'hypometabolism':ab OR 'hypoperfusion':ab OR 'nuclear magnetic resonance imaging'/de OR 'positron emission tomography'/de OR 'single photon emission computed tomography'/de OR ((atrophy NEAR/9 (brain OR matter OR cort* OR neuro*)):ti,ab))
#2a	('parkinson* disease':ti OR 'parkinson* disease':ab OR 'parkinson disease'/de)
#2b	(‘Corticobasal degeneration':ti OR 'CBD':ti OR 'cortical basal ganglionic degeneration':ti OR 'corticobasal syndrome':ti OR 'CBS':ti OR 'corticodentatonigral degeneration with neuronal achromasia':ti OR 'corticonigral degeneration':ti OR 'Corticobasal degeneration':ab OR 'CBD':ab OR 'cortical basal ganglionic degeneration':ab OR 'corticobasal syndrome':ab OR 'CBS':ab OR 'corticodentatonigral degeneration with neuronal achromasia':ab OR 'corticonigral degeneration':ab OR 'corticobasal degeneration'/de)
#2c	('multiple system* atrophy':ti OR 'msa':ti OR 'msa-p':ti OR 'msa-c':ti OR 'olivoponto-cerebellar atrophy':ti OR 'opca':ti OR 'striatonigral degeneration':ti OR 'snd':ti OR 'shy–drager syndrome':ti OR 'multisystem-atrophy':ti OR 'multi-system atrophy':ti OR 'multiple system* atrophy':ab OR 'msa':ab OR 'msa-p':ab OR 'msa-c':ab OR 'olivoponto-cerebellar atrophy':ab OR 'opca':ab OR 'striatonigral degeneration':ab OR 'snd':ab OR 'shy–drager syndrome':ab OR 'multisystem-atrophy':ab OR 'multi-system atrophy':ab OR 'multiple system atrophy'/de)
#2d	('progressive supranuclear pals*':ti,ab OR 'psp':ti,ab OR 'steele-richardson-olszewski':ti,ab OR 'steele richardson olszewski':ti,ab OR 'richardson syndrome':ti,ab OR 'progressive supranuclear palsy'/de)
#3	[embase]/lim

Medline Complete	#1 AND #2
#1	((TI "MRI" OR TI "magnetic resonance imaging" OR AB "MRI" OR AB "magnetic resonance imaging" OR MH “magnetic resonance imaging” OR TI “VBM” OR AB “VBM” OR TI “voxel based morpho*” OR AB “voxel based morpho*” OR TI “SPECT” OR AB “SPECT” OR TI “single photon emission computed tomography” OR AB “single photon emission computed tomography” OR MH “Tomography, Emission-Computed, Single-Photon” OR TI “positron emission tomography” OR AB “positron emission tomography” OR TI “PET” OR AB “PET” OR MH “Positron-Emission Tomography” OR TI “hypometabolism” OR AB “hypometabolism” OR TI “hypoperfusion” OR AB “hypoperfusion”) OR ((TI (atrophy) N9 (brain OR matter OR cort* or neuro*)) OR (AB (atrophy) N9 (brain OR matter OR cort* or neuro*)))
#2a	(TI "parkinson* disease" OR AB "parkinson* disease" OR MH "parkinson disease")
#2b	(TI "Corticobasal degeneration" OR TI "CBD"OR TI "cortical basal ganglionic degeneration" OR TI "corticobasal syndrome" OR TI "CBS" OR TI "corticodentatonigral degeneration with neuronal achromasia" OR TI "corticonigral degeneration" OR AB "Corticobasal degeneration" OR AB "CBD" OR AB "cortical basal ganglionic degeneration" OR AB "corticobasal syndrome" OR AB "CBS" OR AB "corticodentatonigral degeneration with neuronal achromasia" OR AB "corticonigral degeneration" OR MH "corticobasal degeneration")
#2c	(TI "multiple system* atrophy" OR AB "multiple system* atrophy" OR TI "MSA" OR AB "MSA" OR TI "Olivoponto-cerebellar atrophy" OR AB "Olivoponto-cerebellar atrophy" OR TI "OPCA" OR AB "OPCA" OR TI "striatonigral degeneration" OR AB "striatonigral degeneration" OR TI "SND" OR AB "SND" OR TI "MSA-P" OR AB "MSA-P" OR TI "MSA-C" OR AB "MSA-C" OR TI "multisystem-atrophy" OR AB "multisystem-atrophy" OR TI "multi-system atrophy" OR AB "multi-system atrophy" OR TI "Shy–Drager syndrome" OR AB "Shy–Drager syndrome" OR MH "multiple system atrophy")
#2d	(TI "Progressive supranuclear pals*" OR TI "Steele-Richardson-Olszewski" OR TI "Steele Richardson Olszewski" OR TI "Richardson syndrome" OR TI "PSP" OR AB "Progressive supranuclear pals*" OR AB "Steele-Richardson-Olszewski" OR AB "Steele Richardson Olszewski" OR AB "Richardson syndrome" OR AB "PSP" OR MH "progressive supranuclear palsy")

Supplementary Table 2

Included Study Characteristics

Study	Diagnosis	Method	*N* Participants		Mean age (SD) in years		Diagnostic criteria	Diagnostic Certainty (if supplied)
			Patients	Controls	Patients	Controls		
Abe et al., 2003	PD	[123I] IMP SPECT	28	17	67.3 (7.3)	69.6 (10.2)	UKBB	NS
Alzahrani et al., 2016	PD	MRI-VBM	**PD**: 40<br>**PDA**: 25	24	**PD:** 66.1 (8.5)<br>**PDA:** 68.7 (8.4)	62.8 (9.8)	UKBB	NS
Burton et al., 2004	PD	MRI-VBM	**PD**: 31<br>**PDD**: 26	36	**PD:** 75.2 (5.2)<br>**PDD:** 72.3 (5.2)	75.1 (6.6)	UKBB	NS
Camicioli et al., 2009	PD	MRI-VBM	43	43	70.7 (4.0)	71.0 (4.5)	UKBB	NS
Chen et al., 2017	PD	MRI-VBM	**PD-NC**: 11<br>**PD-MCI**: 21	21	**PD-NC:** 59.2 (2.58)<br>**PD-MCI:** 63.6 (11.16)	61.1 (8.3)	UKBB	NS
Chung et al., 2016	PD	FDG-PET	**PD-NC**: 13<br>**PD-MCI**: 11	15	**PD-NC**: 61.5 (7.9)<br>**PD-MCI**: 68.0 (4.6)	65.7 (3.7)	UKBB	NS
Cilia et al., 2008	PD	99MTc-ECD SPECT	**PD**: 40<br>**PDG:** 11	29	**PD**: 55 (7.0)<br>**PDG**: 57.4 (5.8)	56 (6.0)	UKBB	NS
Cordato et al., 2005	PD	MRI-VBM	17	23	67.7 (6.7)	71.5 (7.2)	Gelb Criteria	NS
Fioravanti et al., 2015	PD	MRI-VBM	20	15	60.5 (7.7)	64.6 (4.8)	UKBB	NS
Gama et al., 2014	PD	MRI-VBM	**PD:** 28	10	**PD**: 65.7 (7.8)	68.1 (7.0)		NS

			**PDVH:** 11		**PDVH**: 70.6 (9.1)		NS (clinically confirmed with neurologist)	
Gao et al., 2017	PD	MRI-VBM	**PD:** 23<br>**PD-MCI:** 23	21	**PD**: 62.4 (7.7)<br>**PD-MCI**: 65.1 (8.7)	63.8 (5.4)	UKBB	NS
Gerrits et al., 2014	PD	MRI-VBM	93	46	63.0 (10.)	61.0 (8.0)	UKBB	NS
Guimarães et al., 2017	PD	MRI-VBM	66	40	57.9 (10.3)	57.6 (10.8)	UKBB	NS
Huang et al., 2013	PD	FDG-PET	26	12	66.5 (1.4)	67.4 (2.0)	UKBB	NS
Li et al., 2017	PD	MRI-VBM	366<br>(from PPMI)	172	62.2 (9.8)	60.6 (11.4)	NS (taken from PPMI)	NS
Lin et al., 2013	PD	MRI-VBM	10	13	67.3 (8.8)	65.3 (11.1)	UPDRS	NS
Potgieser et al., 2014	PD	MRI-VBM	77	87	63.0 (10.5)	60.1 (7.2)	UKBB	NS
Ramírez-Ruizet al., 2007	PD	MRI-VBM	**PD:** 20<br>**PDVH:** 18	21	**PD**: NS<br>**PDVH**: NS	NS	UKBB	NS
Summerfield et al., 2005	PD	MRI-VBM	**PD:** 13<br>**PDD:** 16	13	**PD**: 72.8 (4.9)<br>**PDD**: 70.1 (7.9)	70.1 (7.2)	UKBB	NS
Van Laere et al., 2004	PD	[99M]Tc-ECD SPECT	81	44	62.6 (10.2)	59.2 (11.9)	UKBB	NS
Wang et al., 2017	PD	FDG-PET	**PD:** 15	15	**PD:** 64.1 (9.0)	63.3 (4.6)	UKBB	NS

			**PDA:** 13		**PDA**: 68.3 (5.7)			
Zhang et al., 2015	PD	MRI-VBM	**PD:** 14<br>**PD-MCI:** 21	20	**PD**: 58.5 (9.2)<br>**PD-MCI**: 63.8 (8.6)	59.4 (6.4)	UKBB	NS
Hosey et al., 2005	PD	$H_2{}^{15}O$ PET	9	9	59 (9)	53 (12)	UKBB	NS
Hosokai et al., 2009	PD	FDG-PET	**PDNC**: 27<br>**PD-MCI:** 13	13	**PD-NC:** 65.7 (5.1)<br>**PD-MCI:** 67.6 (5.5)	63.0 (4.6)	UKBB	NS
Kostic et al. 2010	PD	MRI-VBM	**PD total**: 40	26	66 (NS) range: 50-79	63 (NS) range: 48-79	UKBB	NS
Lyoo et al., 2010	PD	FDG-PET	**PD total:** 61 **(PD-NC:** 20, **PD-SA:** 12, **PD-SN:** 11, **PD-MD:** 18)	14	**PD total:** 64.0 (NS) range 56-68. **PD-NC:** 62.0 (NS) range: 55.8-73.0. **PD-SA:** 65.5 (NS) range: 56-71. **PD-SN**: 57.0 (NS) range: 54-72. **PD-MD:** 65.5 (NS) range: 60.3-69.	"age matched"	UKBB	NS
Pereira et al., 2012	PD	MRI-VBM	**PD:** 20	20	64 (9.53)	59.1 (10.9)	UKBB	NS
Teune et al., 2010	PD	FDG-PET	20	18	63 (9)	56 (14)	MDS Criteria (Litvan et al., 2003)	NS
Tir et al., 2009	PD	MRI-VBM	19	18	61.6 (7.6)	56(14)	MDS Criteria (Litvan et al., 2003); UKBB	NS
Juh et al., 2004	PD	FDG-PET	8	22	67.9 (10.7)	67.8 (14.4)	UKBB	NS
Gasca-Salas et al., 2016	PD	FDG-PET	**PD-MCI:** 12<br>**PD-MCI+VH:** 9	19	**PD-MCI**: 70.8 (3.4).<br>**PD-MCI+VH**: 70.7(3.9).	70.1 (3.1)	UKBB	NS
Compta et al., 2012	PD	MRI-VBM	**PD-ND:** 18<br>**PDD:** 15	12	**PD-ND**: 69 (NS) range: 67.5-76.75 **PDD**:73 (NS) range: 65-78.	71.5 (NS) range: 67.5-76.75	UKBB	NS

Diez-Cirarda et al., 2015	PD	MRI-VBM	37	15	67.97 (6.17)	71.5 (67.5-76.75)	UKBB	NS
Liang et al., 2016	PD	MRI-VBM	**PD-ND:** 20<br>**PD-DEP:** 16	21	**PD-ND**: 56.43 (6.45)<br>**PD-DEP**: 63.5 (9.87)	65.07 (7.01)	UKBB	NS
Terada et al., 2018	PD	MRI-VBM	40	10	64.7 (8)	67.6 (3.2)	UKBB	NS
Martin et al., 2009	PD	MRI-VBM	26	14	59.8 (7.7)	56.8 (7.8)	Calne et al., 1992	NS
Naduthota et al., 2017	PD	MRI-VBM	72	72	51.4 (10.6)	50.8 (10.4)	UKBB	NS
Nagano-Saito et al., 2005	PD	MRI-VBM	**ND-PD:** 39<br>**ND-PD-Adv.:** 19	31	**ND-PD**: 61.8 (8.1)<br>**ND-PD-Adv.:** 62.6 (7.9)	63.5 (8.8)	Calne et al., 1992	NS
Nobili et al., 2009	PD	^{99m}Tc-ECD SPECT	**PD-MCI:** 15	15	71.5 (+/-5.9).	71.3 (6.1)	Gelb Criteria	NS
Pappatå et al., 2011	PD	FDG-PET	**PD-MCI** 12	12	64 (5.3)	62 (6.2)	UKBB	NS
Song et al., 2014	PD	Tc-99m HMPAO SPECT	**TPD**: 33	33	70.85 (8.65).	66.94 (5.4)	UKBB	NS
Agosta et al., 2013	PD	MRI-VBM	**PD-MOD:** 14<br>**PD-SEV:** 12	42	**PD-MOD**: 65 (8);<br>**PD-SEV:** 65 (7)	64 (7)	UKBB	NS
Pagonabarraga et al., 2014	PD	MRI-VBM	**PD-NH:** 27<br>**PD-mH:** 15	15	**PD-NH:** 66.3 (8);<br>**PD-mH:** 64.1 (9)	66.8 (8)	UKBB	NS
Le Jeune et al., 2010	PD	FDG-PET	20	13	57.9 (9.7)	53.23 (11.2)	UKBB	NS
Lee et al., 2015	PD	MRI-VBM	**PD-L:** 23<br>**PD-R:** 23	23	**PD-L**: 60.7 (6.8).<br>**PD-R**: 57.9 (6.5).	57.9 (6.7)	UKBB	NS
Tessitore et al., 2012	PD	MRI-VBM	**PD-FOG**: 12	12	NS (>45 years)	"age matched"	UKBB	NS
Xuan et al., 2019	PD	MRI-VBM	**EOPD:** 28<br>**M-LOPD:** 37	**Young HC**: 23<br>**Old HC:** 23	**EOPD**: 49.6 (5.9)<br>**M-LOPD**: 62.4 (5.9)	**Young HC**: 51.3 (6.3)<br>**Old HC:** 64.2 (6.7)	UKBB	NS

Kikuchi et al., 2001	PD	Tc-99m HMPAO SPECT	**PD-all:** 18	11	59.1 (11.5)	62 (9.02)	UKBB	NS
Berding et al., 2001	PD	FDG-PET	11	10	56 (10).	49 (16)	NS	NS
Berti et al., 2012	PD	FDG-PET	26	21	65.3 (6.4)	62.4 (9)	Gelb Criteria	NS
Hsu et al., 2007	PD	Tc-99m HMPAO SPECT	21	11	64.4 (8.7).	60.1 (7.5)	UKBB	NS
Imon et al., 1999	PD	^{99m}Tc-ECD SPECT	27	24	65.6 (10).	61.8 (9)	Ward & Gibb Criteria	NS
Jia et al., 2019	PD	MRI-VBM	34	45	**PD-dep:** 59.4 (8.9); **PD-NC:** 59.1 (9.9)	57 (10)	UKBB	NS
Rektorova et al., 2014	PD	MRI-SBM	**PD-NC:** 27 **PD-MCI:**27	25	**PD-NC**: 63.11 (9.27); **PD-MCI**: 62.59 (6.61)	59.44 (5.77)	UKBB	NS
Srivastava et al., 2019	PD	MRI-VBM	**YOPD:** 14	13	NS	NS	UKBB	NS
Yu et al., 2020	PD	MRI-VBM	33	27	63.1 (9.09)	59.2 (9.71)	UKBB	NS
Ruppert et al., 2020	PD	FDG-PET	42	14	67.24 (7.94)	64.50 (8.29)	UKBB	NS
Yang et al., 2019	PD	FDG-PET	**YOPD:** 42	42	42.6 (8.7)	41.6 (6.5)	UKBB	NS
Li et al., 2022	PD	MRI-VBM	84	70	53.31 (10.49)	52.56 (10.92)	UKBB	NS
Liguori et al., 2019	PD	FDG-PET	28	35	65.6 (7.56)	67.89 (4.95)	UKBB	NS
Wu et al., 2020	PD	MRI-VBM	30	15	64.90 (10.08)	62.60 (4.85)	UKBB	NS
Kunst et al., 2019	PD	MRI-VBM	**PD-MCI:** 24	58	65.1 (10)	67.5 (7.3)	MDS Criteria (Postuma et al., 2015; Litvan et al., 2012)	NS
Chu et al., 2019	PD	FDG-PET	50	20	57.7 (11.1)	55.6 (12.3)	UKBB	NS
Ceccarini et al., 2019	PD	MRI-VBM	38	10	63.7 (8.7)	59.1 (11.7)	UKBB	NS
Chen et al., 2019	PD	MRI-VBM	61	59	62.61 (8.59)	60.9 (6.3)	UKBB	NS
Cheng et al., 2020	PD	MRI-VBM	**PDD:** 35	37	64 (5.85)	63.14 (5.34)	Poewe et al., 2008	NS

Furukawa et al., 2020	PD	[^{123}I]IMP SPECT	21	17	66.4 (5.3)	66.7 (9.9)	UKBB	NS
Huang et al., 2019	PD	MRI-VBM	9	7	57.78 (9.05)	56.14 (8.07)	NS	NS
Inguanzo et al., 2021	PD	MRI-VBM	**PD1:** 75; **PD2**: 21	33	**PD1** 75 (NS); **PD2** 68 **(**NS)	66 (NS)	UKBB	NS
Boxer et al., 2006	PSP	MRI-VBM	15	80	70.9 (6.9).	67.9 (8.6)	NINDS-SPSP	probable
Brenneis et al., 2004	PSP	MRI-VBM	12	12	67.5 (6.6)	60 (5.8)	NINDS-SPSP	probable
Cordato et al., 2005	PSP	MRI-VBM	21	23	70.3 (6.4).	71.5 (7.2)	NINDS-SPSP	5 definite; 16 NS
[a]Lagarde et al., 2013	PSP	MRI-VBM	19[a]	18	65.9 (6.5).	67.8 (5.2)	NINDS-SPSP	probable
Lehéricy et al., 2010	PSP	MRI-VBM	10	9	66.9 (6.4)	66.5 (4.8)	NINDS-SPSP	NS
Padovani et al., 2006	PSP	MRI-VBM	14	14	73.0 (5.36).	65.6 (4.1)	NINDS-SPSP	probable
Sakurai et al., 2014	PSP	MRI-VBM	33	32	78 (6).	79 (3).	NINDS-SPSP	4 possible; 29 probable
Takahashi et al., 2011	PSP	MRI-VBM and FDG-PET	16	20	64.6 (6.4).	64.8 (6.4)	NINDS-SPSP	probable
Whitwell et al., 2013	PSP	MRI-VBM	16	20	72.1 (4.6;	73.9 (6.3)	NINDS-SPSP	probable/definite
Agosta et al., 2010	PSP	MRI-VBM	20	24	64.9 (NS) range: 53-82	63.8 (NS) range: 48-79	NINDS-SPSP, Williams et al., 2005	18 probable; 2 possible
Ghosh et al., 2012	PSP	MRI-VBM	23	22	71.1 (8.6).	71.4 (7.6)	MDS Criteria (Litvan et al., 2003)	9 definite; 14 NS
Giordano et al., 2013	PSP	MRI-VBM	15	15	68.91 (1.2).	65.5 (6.1)	NINDS-SPSP	probable
Sandhya et al., 2014	PSP	MRI-VBM	10	8	NS	NS	NINDS-SPSP	probable /possible
Kimura et al., 2011	PSP	^{99m}Tc-ECD SPECT	19	17	73.2 (8)	68.8 (10.7)	NINDS-SPSP	probable /definite
Park et al., 2009	PSP	FDG-PET	14	11	68.9 (6.4)	72 (6)	NINDS-SPSP	probable

Price et al., 2004	PSP	MRI-VBM	12	12	65.3 (5.8).	67.4 (4.6)	MDS Criteria (Litvan et al., 2003)	8 definite; 4 probable
Teune et al., 2010	PSP	FDG-PET	17	18	68 (8)	56 (14)	NINDS-SPSP	13 probable; 4 possible
Varrone et al., 2007	PSP	^{99m}Tc-ECD SPECT	16	10	67 (6)	59 (16)	NINDS-SPSP	12 probable; 4 possible
Wang et al., 2015	PSP	MRI-VBM	24	23	64.17 (6.72)	60.52 (6.47)	NINDS-SPSP	probable /possible
Hosaka et al., 2002	PSP	FDG-PET	**12**	12	62.8 (6.0)	63.8 (7.7)	NINDS-SPSP	probable
Ge et al., 2018	PSP	FDG-PET	**PSP:** 20 **Early stage-PSP**: 14	**HC**: 20 **early stage HC:** 20	**PSP**: 65.3 (8.3); **Early-stage PSP:** 65.6 (8.2)	**HC**: 59.0 (11.4); **Early-stage HC**: 62.5 (6.7)	NINDS-SPSP	probable
Juh et al., 2004	PSP	FDG-PET	7	22	67.6 (4.83)	67.8 (14.4)	Retrospectively clinically diagnosed	NS
Lagarde et al., 2015	PSP	MRI-VBM	20	18	65.5 (6.5)	67.8 (5.2)	NINDS-SPSP	NS
Abe et al., 2016	CBS	^{99m}Tc-ECD SPECT	26	26	76 (5.3).	76.6 (5.8)	Mathew et al., 2012	possible
Albrecht et al., 2019	CBS	MRI-VBM	25	25	66.7 (10.1)	66.2 (10.1)	Otto et al., 2011	NS
Borroni et al., 2008	CBS	MRI-VBM	20	21	62.7 (8)	65.6 (4.1)	Lang, 1994	probable
Boxer et al., 2006	CBS	MRI-VBM	14	80	64.6 (5.9)	67.9 (8.6)	NS	Probable
Pardini et al., 2009	CBS	MRI-VBM	25	14	62 (9)	NS	NS (Neurologist assessment)	NS
Pardini et al., 2019	CBS	FDG-PET	14	13	64.2 (9.5)	61.5 (6.2)	Neurologist assessment and pathological examination (autopsy)	Definite CBD (autopsy)
Sakurai et al., 2014	CBS	MRI-VBM	18	32	79 (5)	79 (3)	Lang, 1994	NS

Spotorno et al., 2015	CBS	MRI-PipeDream	10 (7 scanned)	19	70 (2) *	NS	Armstrong et al., 2013	NS
Teune et al., 2010	CBS	FDG-PET	10	18	39 (9)	56 (14)	Mahapatra et al., 2004	NS
[b]Grossman et al., 2004	CBS	MRI-VBM	9	25	64 (7)	68.5 (9.4)	[b] see note.	NS
Halpern et al., 2004	CBS	MRI-VBM	13 (5 scanned)	12	66.76 (10.15) *	NS	NS (Neurologist assessment)	NS
Hosaka et al., 2002	CBS	FDG-PET	12	12	64.8 (6.3)	63.8 (7.7)	Lang, 1994	probable
Mille et al., 2017	CBS	FDG-PET	29	20	67.4 (7.7)	68 (7.9)	Armstrong et al., 2013 and Boeve 2011	NS
Whitwell et al., 2011	CBS	MRI-VBM	5	20	65 (NS) range: 42–69	63 (NS) range 50–70	Autopsy	definite
Zamboni et al., 2010	CBS	MRI-VBM	31 (26 scanned)	14	65.8 (1.5)*	60.5 (1.9)	Boeve et al., 2003	NS
Misch et al., 2014	CBS	^{99m}Tc-ECD SPECT	31	31	68.5 (1.7)	70 (1.2)	Boeve et al., 2003	NS
Huey et al., 2009	CBS	MRI-VBM	48	14	66 (9)	60 (6) total:62	Boeve et al., 2003	NS
Grimaldi et al., 2019	MSA	FDG-PET	85	60	median: 66 (range: 60.5–71.5 years)	median: 66 (range: NS)	Gilman et al., 2008	probable
Brenneis et al., 2006	MSA	MRI-VBM	**MSA-C:** 13	13	61.3 (6.2)	60.5 (4.4)	Gilman et al., 1999	12 probable; 1 possible
Brenneis et al., 2003	MSA	MRI-VBM	**MSA-P:** 12	12	62 (6.6)	60 (5.8)	Gilman et al., 1999	probable
Chang et al., 2009	MSA	MRI-VBM	**MSA** = 23 [**MSA-C** = 10; **MSA-P=** 13]	37	MSA-C: 57.1 (9.9) MSA-P: 59.8 (8.1)	55.5 (8.6)	Gilman et al., 2008	probable

Dash et al., 2019	MSA	MRI-VBM	**MSA:** 26 **[MSA-C** =18; **MSA-P** = 8; 1 undefined]	25	55.7 (5.4)	55.0 (6.8)	Gilman et al., 2008	probable
El Fakhri et al., 2006	MSA	^{99m}Tc-ECD SPECT	5	9	66.8 (11.5)	63.8 (8.1)	Gilman et al., 1999	probable
Fiorenzato et al., 2017	MSA	MRI-VBM	72	36	63.8 (6.8)	61.6 (7.4)	Gilman et al., 2008	probable
Juh et al., 2005	MSA	FDG-PET	11	22	58.5 (8.4)	67.8 (14.4)	Quinn Criteria (Wenning et al., 1994)	probable
Kimura et al., 2011	MSA	^{99m}Tc-ECD SPECT	**MSA-P**: 12	17	69.3 (7.3)	68.8 (10.7)	Gilman et al., 1999	probable
Lee et al., 2008	MSA	FDG-PET	**MSA-C:** 41	30	56.9 (6.9)	55.2 (5.8)	Gilman et al., 1999	probable
Minnerop et al., 2010	MSA	MRI-VBM	14	14	61.1 (3.3)	58.6 (5.1)	Gilman et al., 1999	NS
Shigemoto et al., 2013	MSA	MRI-VBM	**MSA-P:** 20	30	62.9 (7.7)	62.9 (7.7)	Gilman et al., 2008	16 probable; 4 possible
Specht et al., 2003	MSA	MRI-VBM	**MSA-C:** 14	13	59.4 (7.4)	55.1 (6.9)	Gilman et al., 1999	9 probable; 5 possible
Teune et al., 2010	MSA	FDG-PET	21	18	64 (10)	56 (14)	Gilman et al., 2008	13 probable MSA-P; 1 probable MSA-C; 7 possible MSA-P
Tir et al., 2009	MSA	MRI-VBM	**MSA-P:** 14	14	63.6 (9.74)	59.2 (7.6)	Gilman et al., 1999	probable
Tzarouchi et al., 2010	MSA	MRI-VBM	**MSA-P:** 11	11	61.9 (11.7)	64.63 (10.4)	Gilman et al., 2008	NS

Van Laere et al., 2004	MSA	^{99m}Tc-ECD SPECT	15	44	61.4 (9.2)	59.2 (11.9)	Quinn Criteria (Wenning et al., 1994)	probable
Planetta et al., 2015	MSA	MRI-VBM	**MSA-P:** 14	14	64.6 (9)	61.9 (8.4)	Gilman et al., 2008	probable
Minnerop et al., 2007	MSA	MRI-VBM	**Total:** 48 [**MSA-C**: 32 **MSA-P**: 16]	**Total**: 46 **Subset:** (matched to MSA-P): 16	61.2 (6)	58.7 (6.1) **Subset** 62.3 (4.3)	Gilman et al., 1999	8 possible; 24 probable
Shen et al., 2020	MSA	FDG-PET	**MSA-P:** 20	20	62.3 (8.8)	61.7 (7.4)	Gilman et al., 2008	probable
Cao et al., 2021	MSA	MRI-VBM	**MSA-NCI:** 25**;** **MSA-MCI:** 20;	29	**MSA-NCI:** 62.52 (6.90); **MSA-MCI:** 64.15 (9.22)	61.17 (6.77)	Gilman et al., 2008	probable
Kawabata et al., 2019	MSA	MRI-VBM	32	32	63.4 (7.8)	63.4 (7.6)	Gilman et al., 2008	probable
Lyoo et al., 2008	MSA	FDG-PET	37	16	61 (NS)	61 (NS)	Gilman et al., 1999	possible and probable

Note. Authors in italics represent studies that were qualitatively analysed where contrasts did not include more than 10 studies for meta-analysis. All studies in this table were included in the parkinsonian disorder combined cohort meta-analysis. SD = standard deviation; UKBB = United Kingdom Brain Bank criteria for Parkinson's disease; PD = Idiopathic Parkinson's disease; PSP = Progressive Supranuclear Palsy; CBS = Cortiobasal Syndrome; MSA = Multiple System Atrophy; HC = Healthy controls; PD-NC = Parkinson's disease normal cognitive functioning; PD-MCI = Parkinson's disease mild cognitive impairment; PDD = Parkinson's disease dementia; PD-mH = Parkinson's disease -minor hallucinations; PD-NH = Parkinson's disease-without hallucinations; PD-MILD = Parkinson's disease mild severity; PD-MOD = Parkinson's disease moderate severity; PD-SEV = Parkinson's disease severe severity; PD-FOG = Parkinson's disease with freezing of gait; EOPD = early-onset Parkinson's disease; LOPD = late-onset Parkinson's disease; YOPD = young-onset Parkinson's disease; PD-ICD = Parkinson's disease impulse control disorder; ND-PD = non-demented Parkinson's disease; ND-PD-adv = non-demented advanced Parkinson's disease; PD-ND = Parkinson's disease without depression; PD-DEP = Parkinson's disease with depression; PD-MCI+VH = Parkinson's disease mild cognitive impairment with visual hallucinations; PD-SA = Parkinson's disease single amnestic; PD-SN = Parkinson's disease single non-amnestic mild cognitive impairment; PD-MD = Parkinson's disease multiple domains; PDG = Parkinson's disease with pathological gambling; PDA = Parkinson's disease apathy; TPD = Tremor dominant Parkinson's disease; PD-L = Parkinson's disease left; PD-R = Parkinson's disease right; MSA-C = Multiple System Atrophy-Cerebellar; MSA-P = Multiple System Atrophy-Parkinsonism; NS = Not Specified; VBM = voxel-based morphometry; SBM = Source-based morphometry; [a] Lagarde et al. 2013 - 1 patient did not undergo scanning but age of that patient was included in the M and SD calculations. [b]Grossman et al. nine patients were given the clinical diagnosis of CBD based on clinical-pathological studies reported in the literature and authors' own autopsy series (Rinne et al., 1994; Grimes et al., 1999; Riley and Lang 2000; Forman et al., 2002). [c]Dash et al. was not included in the MSA-P meta-analysis as there were no significant differences reported between MSA-P patients and controls, diagnosis information of the total cohort is provided here. *denotes records where it is not clear whether non-scanned participants were included in the calculation of the demographic averages.

Supplementary References (Supplementary Table 2)

Abe, Y., Kachi, T., Kato, T., Arahata, Y., Yamada, T., Washimi, Y., Iwai, K., Ito, K., Yanagisawa, N., & Sobue, G. (2003). Occipital hypoperfusion in Parkinson's disease without dementia: Correlation to impaired cortical visual processing. *Journal of Neurology Neurosurgery and Psychiatry*, *74*(4), 419–422. https://doi.org/10.1136/jnnp.74.4.419

Abe, Y., Kimura, N., Goto, M., Aso, Y., & Matsubara, E. (2016). Brain Perfusion in Corticobasal Syndrome with Progressive Aphasia. *Dementia and Geriatric Cognitive Disorders Extra*, *6*(1), 133–141. https://doi.org/10.1159/000443329

Agosta, F., Canu, E., Stojković, T., Pievani, M., Tomić, A., Sarro, L., Dragašević, N., Copetti, M., Comi, G., Kostić, V. S., & Filippi, M. (2013). The topography of brain damage at different stages of parkinson's disease. *Human Brain Mapping*, *34*(11), 2798–2807. https://doi.org/10.1002/hbm.22101

Agosta, F., Kostić, V. S., Galantucci, S., Mesaroš, Š., Svetel, M., Pagani, E., Stefanova, E., & Filippi, M. (2010). The in vivo distribution of brain tissue loss in Richardson's syndrome and PSP-parkinsonism: A VBM-DARTEL study. *European Journal of Neuroscience*, *32*(4), 640–647. https://doi.org/10.1111/j.1460-9568.2010.07304.x

Albrecht, F., Mueller, K., Ballarini, T., Lampe, L., Diehl-Schmid, J., Fassbender, K., Fliessbach, K., Jahn, H., Jech, R., Kassubek, J., Kornhuber, J., Landwehrmeyer, B., Lauer, M., Ludolph, A. C., Lyros, E., Prudlo, J., Schneider, A., Synofzik, M., Wiltfang, J., … Schroeter, M. L. (2019). Unraveling corticobasal syndrome and alien limb syndrome with structural brain imaging. *Cortex*, *117*, 33–40. https://doi.org/10.1016/j.cortex.2019.02.015

Alzahrani, H., Antonini, A., & Venneri, A. (2016). Apathy in Mild Parkinson's Disease: Neuropsychological and Neuroimaging Evidence. *Journal of Parkinson's Disease*, *6*(4), 821–832. https://doi.org/10.3233/JPD-160809

Berding, G., Odin, P., Brooks, D. J., Nikkhah, G., Matthies, C., Peschel, T., Shing, M., Kolbe, H., Hoff, J. van den, Fricke, H., Dengler, R., Samii, M., & Knapp, W. H. (2001). Resting Regional Cerebral Glucose Metabolism in Advanced Parkinson's Disease Studied in the Offand On Conditions with [18F]FDG-PET. *Movement Disorders*, *16*(6), 1007–1013. https://doi.org/10.1002/mds.1221

Berti, V., Polito, C., Borghammer, P., Ramat, S., Mosconi4, L., E. Vanzi1, M. T. De, Cristofaro1, M., De Leon, S., Sorbi, & Pupi, A. (2011). Alternative normalization methods demonstrate widespread cortical hypometabolism in untreated de novo Parkinson's disease. *J Nucl Med Mol Imaging*, *23*(1), 1–7. https://www.ncbi.nlm.nih.gov/pmc/articles/PMC3624763/pdf/nihms412728.pdf

Borroni, B., Garibotto, V., Agosti, C., Brambati, S. M., Bellelli, G., Gasparotti, R., Padovani, A., & Perani, D. (2008). White matter changes in corticobasal degeneration syndrome and correlation with limb apraxia. *Archives of Neurology*, *65*(6), 796–801. https://doi.org/10.1001/archneur.65.6.796

Boxer, A. L., Geschwind, M. D., Belfor, N., Gorno-Tempini, M. L., Schauer, G. F., Miller, B. L., Weiner, M. W., & Rosen, H. J. (2006). Patterns of brain atrophy that differentiate corticobasal degeneration syndrome from progressive supranuclear palsy. *Archives of Neurology, 63*(1), 81–86. https://doi.org/10.1001/archneur.63.1.81

Boxer, A. L., Geschwind, M. D., Belfor, N., Gorno-Tempini, M. L., Schauer, G. F., Miller, B. L., Weiner, M. W., & Rosen, H. J. (2006). Patterns of brain atrophy that differentiate corticobasal degeneration syndrome from progressive supranuclear palsy. *Archives of Neurology, 63*(1), 81–86. https://doi.org/10.1001/archneur.63.1.81

Brenneis, C., Boesch, S. M., Egger, K. E., Seppi, K., Scherfler, C., Schocke, M., Wenning, G. K., & Poewe, W. (2006). Cortical atrophy in the cerebellar variant of multiple system atrophy: A voxel-based morphometry study. *Movement Disorders*, *21*(2), 159–165. https://doi.org/10.1002/mds.20656

Brenneis, C., Seppi, K., Schocke, M. F., Müller, J., Luginger, E., Bösch, S., Löscher, W. N., Büchel, C., Poewe, W., & Wenning, G. K. (2003). Voxel-based morphometry detects cortical atrophy in the Parkinson variant of multiple system atrophy. *Movement Disorders*, *18*(10), 1132–1138. https://doi.org/10.1002/mds.10502

Brenneis, C., Seppi, K., Schocke, M., Benke, T., Wenning, G. K., & Poewe, W. (2004). Voxel based morphometry reveals a distinct pattern of frontal atrophy in progressive supranuclear palsy. *Journal of Neurology, Neurosurgery and Psychiatry, 75*(2), 246–249. https://doi.org/10.1136/jnnp.2003.015297

Burton, E. J., McKeith, I. G., Burn, D. J., Williams, E. D., & O'Brien, J. T. (2004). Cerebral atrophy in Parkinson's disease with and without dementia: A comparison with Alzheimer's disease, dementia with Lewy bodies and controls. *Brain*, *127*(4), 791–800. https://doi.org/10.1093/brain/awh088

Camicioli, R., Gee, M., Bouchard, T. P., Fisher, N. J., Hanstock, C. C., Emery, D. J., & Martin, W. R. W. (2009). Voxel-based morphometry reveals extra-nigral atrophy patterns associated with dopamine refractory cognitive and motor impairment in parkinsonism. *Parkinsonism and Related Disorders*, *15*(3), 187–195. https://doi.org/10.1016/j.parkreldis.2008.05.002

Cao, C., Wang, Q., Yu, H., Yang, H., Li, Y., Guo, M., Huo, H., & Fan, G. (2021). Morphological Changes in Cortical and Subcortical Structures in Multiple System Atrophy Patients With Mild Cognitive Impairment. *Frontiers in Human Neuroscience, 15*(March), 1–8. https://doi.org/10.3389/fnhum.2021.649051

Ceccarini, J., Casteels, C., Ahmad, R., Crabbé, M., Van de Vliet, L., Vanhaute, H., Vandenbulcke, M., Vandenberghe, W., & Van Laere, K. (2019). Regional changes in the type 1 cannabinoid receptor are associated with cognitive dysfunction in Parkinson's disease. *European Journal of Nuclear Medicine and Molecular Imaging*, *46*(11), 2348–2357. https://doi.org/10.1007/s00259-019-04445-x

Chang, C. C., Chang, Y. Y., Chang, W. N., Lee, Y. C., Wang, Y. L., Lui, C. C., Huang, C. W., & Liu, W. L. (2009). Cognitive deficits in multiple system atrophy correlate with

frontal atrophy and disease duration. *European Journal of Neurology*, *16*(10), 1144–1150. https://doi.org/10.1111/j.1468-1331.2009.02661.x

Chen, B., Wang, S., Sun, W., Shang, X., Liu, H., Liu, G., Gao, J., & Fan, G. (2017). Functional and structural changes in gray matter of parkinson's disease patients with mild cognitive impairment. *European Journal of Radiology*, *93*(May), 16–23. https://doi.org/10.1016/j.ejrad.2017.05.018

Chen, Y. S., Chen, H. L., Lu, C. H., Lee, C. Y., Chou, K. H., Chen, M. H., Yu, C. C., Lai, Y. R., Chiang, P. L., & Lin, W. C. (2021). The corticolimbic structural covariance network as an early predictive biosignature for cognitive impairment in Parkinson's disease. *Scientific Reports*, *11*(1). https://doi.org/10.1038/s41598-020-79403-x

Cheng, L., Wu, X., Guo, R., Wang, Y., Wang, W., He, P., Lin, H., & Shen, J. (2020). Discriminative pattern of reduced cerebral blood flow in Parkinson's disease and Parkinsonism-Plus syndrome: An ASL-MRI study. *BMC Medical Imaging*, *20*(1), 1–9. https://doi.org/10.1186/s12880-020-00479-y

Chu, J. S., Liu, T. H., Wang, K. L., Han, C. L., Liu, Y. P., Michitomo, S., Zhang, J. G., Fang, T., & Meng, F. G. (2019). The metabolic activity of caudate and prefrontal cortex negatively correlates with the severity of idiopathic Parkinson's disease. *Aging and Disease*, *10*(4), 847–853. https://doi.org/10.14336/AD.2018.0814

Chung, E. J., Han, Y. H., Mun, C. W., Bae, S. K., Lee, S. M., Jeong, H. W., & Kim, S. J. (2016). Hypometabolism based on a cutoff point on the mini-mental state examination in Parkinson's disease. *Neurology Asia*, *21*(3), 247–253.

Cilia, R., Siri, C., Marotta, G., Isaias, I. U., De Gaspari, D., Canesi, M., Pezzoli, G., & Antonini, A. (2008). Functional abnormalities underlying pathological gambling in parkinson disease. *Archives of Neurology*, *65*(12), 1604–1611. https://doi.org/10.1001/archneur.65.12.1604

Compta, Y., Ibarretxe-Bilbao, N., Pereira, J. B., Junqué, C., Bargalló, N., Tolosa, E., Valldeoriola, F., Muñoz, E., Camara, A., Buongiorno, M., & Martí, M. J. (2012). Grey matter volume correlates of cerebrospinal markers of Alzheimer-pathology in Parkinson's disease and related dementia. *Parkinsonism and Related Disorders*, *18*(8), 941–947. https://doi.org/10.1016/j.parkreldis.2012.04.028

Cordato, N. J., Duggins, A. J., Halliday, G. M., Morris, J. G. L., & Pantelis, C. (2005). Clinical deficits correlate with regional cerebral atrophy in progressive supranuclear palsy. *Brain*, *128*(6), 1259–1266. https://doi.org/10.1093/brain/awh508

Cordato, N. J., Duggins, A. J., Halliday, G. M., Morris, J. G. L., & Pantelis, C. (2005). Clinical deficits correlate with regional cerebral atrophy in progressive supranuclear palsy. *Brain*, *128*(6), 1259–1266. https://doi.org/10.1093/brain/awh508

Dash, S. K., Stezin, A., Takalkar, T., George, L., Kamble, N. L., Netravathi, M., Yadav, R., Kumar, K. J., Ingalhalikar, M., Saini, J., & Pal, P. K. (2019). Abnormalities of white and grey matter in early multiple system atrophy: comparison of parkinsonian and cerebellar

variants. *European Radiology*, *29*(2), 716–724. https://doi.org/10.1007/s00330-018-5594-9

Díez-Cirarda, M., Ojeda, N., Peña, J., Cabrera-Zubizarreta, A., Gómez-Beldarrain, M. Á., Gómez-Esteban, J. C., & Ibarretxe-Bilbao, N. (2015). Neuroanatomical correlates of theory of mind deficit in Parkinson’s disease: A multimodal imaging study. *PLoS ONE*, *10*(11), 1–16. https://doi.org/10.1371/journal.pone.0142234

El Fakhri, G., Habert, M. O., Maksud, P., Kas, A., Malek, Z., Kijewski, M. F., & Lacomblez, L. (2006). Quantitative simultaneous 99mTc-ECD/123I-FP-CIT SPECT in Parkinson’s disease and multiple system atrophy. *European Journal of Nuclear Medicine and Molecular Imaging*, *33*(1), 87–92. https://doi.org/10.1007/s00259-005-1920-y

Fioravanti, V., Benuzzi, F., Codeluppi, L., Contardi, S., Cavallieri, F., Nichelli, P., & Valzania, F. (2015). MRI correlates of Parkinson’s disease progression: A voxel based morphometry study. *Parkinson’s Disease*, *2015*. https://doi.org/10.1155/2015/378032

Fiorenzato, E., Weis, L., Seppi, K., Onofrj, M., Cortelli, P., Zanigni, S., Tonon, C., Kaufmann, H., Shepherd, T. M., Poewe, W., Krismer, F., Wenning, G., Antonini, A., & Biundo, R. (2017). Brain structural profile of multiple system atrophy patients with cognitive impairment. *Journal of Neural Transmission*, *124*(3), 293–302. https://doi.org/10.1007/s00702-016-1636-0

Furukawa, S., Hirano, S., Yamamoto, T., Asahina, M., Uchiyama, T., Yamanaka, Y., Nakano, Y., Ishikawa, A., Kojima, K., Abe, M., Uji, Y., Higuchi, Y., Horikoshi, T., Uno, T., & Kuwabara, S. (2020). Decline in drawing ability and cerebral perfusion in Parkinson’s disease patients after subthalamic nucleus deep brain stimulation surgery. *Parkinsonism and Related Disorders*, *70*(December), 60–66. https://doi.org/10.1016/j.parkreldis.2019.12.002

Gama, R. L., Bruin, V. M. S., Távora, D. G. F., Duran, F. L. S., Bittencourt, L., & Tufik, S. (2014). Structural brain abnormalities in patients with Parkinson’s disease with visual hallucinations: A comparative voxel-based analysis. *Brain and Cognition*, *87*(1), 97–103. https://doi.org/10.1016/j.bandc.2014.03.011

Gao, Y., Nie, K., Huang, B., Mei, M., Guo, M., Xie, S., Huang, Z., Wang, L., Zhao, J., Zhang, Y., & Wang, L. (2017). Changes of brain structure in Parkinson’s disease patients with mild cognitive impairment analyzed via VBM technology. *Neuroscience Letters*, *658*(May), 121–132. https://doi.org/10.1016/j.neulet.2017.08.028

Gasca-Salas, C., Clavero, P., García-García, D., Obeso, J. A., & Rodríguez-Oroz, M. C. (2016). Significance of visual hallucinations and cerebral hypometabolism in the risk of dementia in Parkinson’s disease patients with mild cognitive impairment. *Human Brain Mapping*, *37*(3), 968–977. https://doi.org/10.1002/hbm.23080

Ge, J., Wu, J., Peng, S., Wu, P., Wang, J., Zhang, H., Guan, Y., Eidelberg, D., Zuo, C., & Ma, Y. (2018). Reproducible network and regional topographies of abnormal glucose metabolism associated with progressive supranuclear palsy: Multivariate and univariate analyses in American and Chinese patient cohorts. *Human Brain Mapping*, *39*(7), 2842–2858. https://doi.org/10.1002/hbm.24044

Gerrits, N. J. H. M., van der Werf, Y. D., Hofman, M., Foncke, E. M. J., Klein, M., Berendse, H. W., & van den Heuvel, O. A. (2014). Gray matter differences contribute to variation in cognitive performance in Parkinson's disease. *European Journal of Neurology*, *21*(2), 245–252. https://doi.org/10.1111/ene.12269

Ghosh, B. C. P., Calder, A. J., Peers, P. V., Lawrence, A. D., Acosta-Cabronero, J., Pereira, J. M., Hodges, J. R., & Rowe, J. B. (2012). Social cognitive deficits and their neural correlates in progressive supranuclear palsy. *Brain*, *135*(7), 2089–2102. https://doi.org/10.1093/brain/aws128

Giordano, A., Tessitore, A., Corbo, D., Cirillo, G., de Micco, R., Russo, A., Liguori, S., Cirillo, M., Esposito, F., & Tedeschi, G. (2013). Clinical and cognitive correlations of regional gray matter atrophy in progressive supranuclear palsy. *Parkinsonism and Related Disorders*, *19*(6), 590–594. https://doi.org/10.1016/j.parkreldis.2013.02.005

Grimaldi, S., Boucekine, M., Witjas, T., Fluchère, F., Renaud, M., Azulay, J. P., Guedj, E., & Eusebio, A. (2019). Multiple System Atrophy: Phenotypic spectrum approach coupled with brain 18-FDG PET. *Parkinsonism and Related Disorders*, *67*(September), 3–9. https://doi.org/10.1016/j.parkreldis.2019.09.005

Grossman, M., McMillan, C., Moore, P., Ding, L., Glosser, G., Work, M., & Gee, J. (2004). What's in a name: Voxel-based morphometric analyses of MRI and naming difficulty in Alzheimer's disease, frontotemporal dementia and corticobasal degeneration. *Brain*, *127*(3), 628–649. https://doi.org/10.1093/brain/awh075

Guimarães, R. P., Santos, M. C. A., Dagher, A., Campos, L. S., Azevedo, P., Piovesana, L. G., De Campos, B. M., Larcher, K., Zeighami, Y., Amato-Filho, A. C. S., Cendes, F., & Frota D'Abreu, A. C. (2017). Pattern of reduced functional connectivity and structural abnormalities in Parkinson's disease: An exploratory study. *Frontiers in Neurology*, *7*. https://doi.org/10.3389/fneur.2016.00243

Halpern, C. H., Glosser, G., Clark, R., Gee, J., Moore, P., Dennis, K., McMillan, C., Colcher, A., & Grossman, M. (2004). Dissociation of numbers and objects in corticobasal degeneration and semantic dementia. *Neurology*, *62*(7), 1163–1169. https://doi.org/10.1212/01.WNL.0000118209.95423.96

Hosaka, K., Ishii, K., Sakamoto, S., Mori, T., Sasaki, M., Hirono, N., & Mori, E. (2002). Voxel-based comparison of regional cerebral glucose metabolism between PSP and corticobasal degeneration. *Journal of the Neurological Sciences*, *199*(1–2), 67–71. https://doi.org/10.1016/S0022-510X(02)00102-8

Hosaka, K., Ishii, K., Sakamoto, S., Mori, T., Sasaki, M., Hirono, N., & Mori, E. (2002). Voxel-based comparison of regional cerebral glucose metabolism between PSP and corticobasal degeneration. *Journal of the Neurological Sciences*, *199*(1–2), 67–71. https://doi.org/10.1016/S0022-510X(02)00102-8

Hosey, L. A., Thompson, J. L. W., Verhagen Metman, L., Van Den Munckhof, P., & Braun, A. R. (2005). Temporal dynamics of cortical and subcortical responses to apomorphine in Parkinson disease: An H215O PET study. *Clinical Neuropharmacology*, *28*(1), 18–27. https://doi.org/10.1097/01.wnf.0000154220.30263.0e

Hosokai, Y., Nishio, Y., Hirayama, K., Takeda, A., Ishioka, T., Sawada, Y., Suzuki, K., Itoyama, Y., Takahashi, S., Fukuda, H., & Mori, E. (2009). Distinct patterns of regional cerebral glucose metabolism in Parkinson's disease with and without mild cognitive impairment. *Movement Disorders*, *24*(6), 854–862. https://doi.org/10.1002/mds.22444

Hsu, J. L., Jung, T. P., Hsu, C. Y., Hsu, W. C., Chen, Y. K., Duann, J. R., Wang, H. C., & Makeig, S. (2007). Regional CBF changes in Parkinson's disease: A correlation with motor dysfunction. *European Journal of Nuclear Medicine and Molecular Imaging*, *34*(9), 1458–1466. https://doi.org/10.1007/s00259-006-0360-7

Huang, C., Ravdin, L. D., Nirenberg, M. J., Piboolnurak, P., Severt, L., Maniscalco, J. S., Solnes, L., Dorfman, B. J., & Henchcliffe, C. (2013). Neuroimaging markers of motor and nonmotor features of parkinson's disease: An [18F]fluorodeoxyglucose positron emission computed tomography study. *Dementia and Geriatric Cognitive Disorders*, *35*(3–4), 183–196. https://doi.org/10.1159/000345987

Huang, L. C., Wu, P. A., Lin, S. Z., Pang, C. Y., & Chen, S. Y. (2019). Graph theory and network topological metrics may be the potential biomarker in Parkinson's disease. *Journal of Clinical Neuroscience*, *68*, 235–242. https://doi.org/10.1016/j.jocn.2019.07.082

Huey, E. D., Pardini, M., Cavanagh, A., Wassermann, E. M., Kapogiannis, D., Spina, S., Ghetti, B., & Grafman, J. (2009). Association of ideomotor apraxia with frontal gray matter volume loss in corticobasal syndrome. *Archives of Neurology*, *66*(10), 1274–1280. https://doi.org/10.1001/archneurol.2009.218

Imon, Y., Matsuda, H., Ogawa, M., Kogure, D., & Sunohara, N. (1999). SPECT image analysis using statistical parametric mapping in patients with Parkinson's disease. *Journal of Nuclear Medicine*, *40*(10), 1583–1589.

Inguanzo, A., Sala-llonch, R., Segura, B., Erostarbe, H., & Abos, A. (2020). *Parkinsonism and Related Disorders Hierarchical cluster analysis of multimodal imaging data identifies brain atrophy and cognitive patterns in Parkinson ' s disease*. *January*.

Jia, X., Li, Y., Li, K., Liang, P., & Fu, X. (2019). Precuneus dysfunction in Parkinson's disease with mild cognitive impairment. *Frontiers in Aging Neuroscience*, *11*(JAN), 1–9. https://doi.org/10.3389/fnagi.2018.00427

Juh, R., Kim, J., Moon, D., Choe, B., & Suh, T. (2004). Different metabolic patterns analysis of Parkinsonism on the 18F-FDG PET. *European Journal of Radiology*, *51*(3), 223–233. https://doi.org/10.1016/S0720-048X(03)00214-6

Juh, R., Kim, J., Moon, D., Choe, B., & Suh, T. (2004). Different metabolic patterns analysis of Parkinsonism on the 18F-FDG PET. *European Journal of Radiology*, *51*(3), 223–233. https://doi.org/10.1016/S0720-048X(03)00214-6

Juh, R., Pae, C. U., Lee, C. U., Yang, D., Chung, Y., Suh, T., & Choe, B. (2005). Voxel based comparison of glucose metabolism in the differential diagnosis of the multiple system atrophy using statistical parametric mapping. *Neuroscience Research*, *52*(3), 211–219. https://doi.org/10.1016/j.neures.2005.03.010

Kawabata, K., Hara, K., Watanabe, H., Bagarinao, E., Ogura, A., Masuda, M., Yokoi, T., Kato, T., Ohdake, R., Ito, M., Katsuno, M., & Sobue, G. (2019). Alterations in Cognition-Related Cerebello-Cerebral Networks in Multiple System Atrophy. *Cerebellum*, *18*(4), 770–780. https://doi.org/10.1007/s12311-019-01031-7

Kikuchi, A., Takeda, A., Kimpara, T., Nakagawa, M., Kawashima, R., Sugiura, M., Kinomura, S., Fukuda, H., Chida, K., Okita, N., Takase, S., & Itoyama, Y. (2001). Hypoperfusion in the supplementary motor area, dorsolateral prefrontal cortex and insular cortex in Parkinson's disease. *Journal of the Neurological Sciences*, *193*(1), 29–36. https://doi.org/10.1016/S0022-510X(01)00641-4

Kimura, N., Hanaki, S., Masuda, T., Hanaoka, T., Hazama, Y., Okazaki, T., Arakawa, R., & Kumamoto, T. (2011). Brain perfusion differences in Parkinsonian disorders. *Movement Disorders*, *26*(14), 2530–2537. https://doi.org/10.1002/mds.23915

Kimura, N., Hanaki, S., Masuda, T., Hanaoka, T., Hazama, Y., Okazaki, T., Arakawa, R., & Kumamoto, T. (2011). Brain perfusion differences in Parkinsonian disorders. *Movement Disorders*, *26*(14), 2530–2537. https://doi.org/10.1002/mds.23915

Kostić, V. S., Agosta, F., Petrović, I., Galantucci, S., Špica, V., Ječmenica-Lukic, M., & Filippi, M. (2010). Regional patterns of brain tissue loss associated with depression in Parkinson disease. *Neurology*, *75*(10), 857–863. https://doi.org/10.1212/WNL.0b013e3181f11c1d

Kunst, J., Marecek, R., Klobusiakova, P., Balazova, Z., Anderkova, L., Nemcova-Elfmarkova, N., & Rektorova, I. (2019). Patterns of Grey Matter Atrophy at Different Stages of Parkinson's and Alzheimer's Diseases and Relation to Cognition. *Brain Topography*, *32*(1), 142–160. https://doi.org/10.1007/s10548-018-0675-2

Lagarde, J., Valabrégue, R., Corvol, J. C., Garcin, B., Volle, E., Le Ber, I., Vidailhet, M., Dubois, B., & Levy, R. (2015). Why do patients with neurodegenerative frontal syndrome fail to answer: "In what way are an orange and a banana alike?" *Brain*, *138*(2), 456–471. https://doi.org/10.1093/brain/awu359

Lagarde, J., Valabrègue, R., Corvol, J. C., Pineau, F., Le Ber, I., Vidailhet, M., Dubois, B., & Levy, R. (2013). Are frontal cognitive and atrophy patterns different in PSP and bvFTD? A comparative neuropsychological and VBM study. *PLoS ONE*, *8*(11), 1–10. https://doi.org/10.1371/journal.pone.0080353

Le Jeune, F., Péron, J., Grandjean, D., Drapier, S., Haegelen, C., Garin, E., Millet, B., & Vérin, M. (2010). Subthalamic nucleus stimulation affects limbic and associative circuits: A PET study. *European Journal of Nuclear Medicine and Molecular Imaging*, *37*(8), 1512–1520. https://doi.org/10.1007/s00259-010-1436-y

Lee, E.-Y., Sen, S., Eslinger, P. J., Wagner, D., Kong, L., Lewis, M. M., Du, G., & Huang, X. (2017). Side of Motor Onset is Associated with Hemisphere-Specific Memory Decline and Lateralized Gray Matter Loss in Parkinson's disease. *Physiology & Behavior*, *176*(3), 139–148. https://doi.org/10.1016/j.parkreldis.2015.02.008.Side

Lee, P. H., An, Y. S., Yong, S. W., & Yoon, S. N. (2008). Cortical metabolic changes in the cerebellar variant of multiple system atrophy: A voxel-based FDG-PET study in 41 patients. *NeuroImage*, *40*(2), 796–801. https://doi.org/10.1016/j.neuroimage.2007.11.055

Lehéricy, S., Hartmann, A., Lannuzel, A., Galanaud, D., Delmaire, C., Bienaimée, M. J., Jodoin, N., Roze, E., Gaymard, B., & Vidailhet, M. (2010). Magnetic resonance imaging lesion pattern in Guadeloupean parkinsonism is distinct from progressive supranuclear palsy. *Brain*, *133*(8), 2410–2425. https://doi.org/10.1093/brain/awq162

Li, R., Zou, T., Wang, X., Wang, H., Hu, X., Xie, F., Meng, L., & Chen, H. (2022). Basal ganglia atrophy–associated causal structural network degeneration in Parkinson's disease. *Human Brain Mapping*, *43*(3), 1145–1156. https://doi.org/10.1002/hbm.25715

Li, X., Xing, Y., Schwarz, S. T., & Auer, D. P. (2017). Limbic grey matter changes in early Parkinson's disease. *Human Brain Mapping*, *38*(7), 3566–3578. https://doi.org/10.1002/hbm.23610

Liang, P., Deshpande, G., Zhao, S., Liu, J., Hu, X., & Li, K. (2016). Altered directional connectivity between emotion network and motor network in Parkinson's disease with depression. *Medicine (United States)*, *95*(30). https://doi.org/10.1097/MD.0000000000004222

Liguori, C., Ruffini, R., Olivola, E., Chiaravalloti, A., Izzi, F., Stefani, A., Pierantozzi, M., Mercuri, N. B., Modugno, N., Centonze, D., Schillaci, O., & Placidi, F. (2019). Cerebral glucose metabolism in idiopathic REM sleep behavior disorder is different from tau-related and α-synuclein-related neurodegenerative disorders: A brain [18F]FDG PET study. *Parkinsonism and Related Disorders*, *64*(September 2018), 97–105. https://doi.org/10.1016/j.parkreldis.2019.03.017

Lin, C. H., Chen, C. M., Lu, M. K., Tsai, C. H., Chiou, J. C., Liao, J. R., & Duann, J. R. (2013). VBM Reveals Brain Volume differences between Parkinson's disease and essential tremor patients. *Frontiers in Human Neuroscience*, *di*(MAY), 1–10. https://doi.org/10.3389/fnhum.2013.00247

Lyoo, C. H., Jeong, Y., Ryu, Y. H., Lee, S. Y., Song, T. J., Lee, J. H., Rinne, J. O., & Lee, M. S. (2008). Effects of disease duration on the clinical features and brain glucose metabolism in patients with mixed type multiple system atrophy. *Brain*, *131*(2), 438–446. https://doi.org/10.1093/brain/awm328

Lyoo, C. H., Jeong, Y., Ryu, Y. H., Rinne, J. O., & Lee, M. S. (2010). Cerebral glucose metabolism of Parkinson's disease patients with mild cognitive impairment. *European Neurology*, *64*(2), 65–73. https://doi.org/10.1159/000315036

Martin, W. R. W., Wieler, M., Gee, M., & Camicioli, R. (2009). Temporal lobe changes in early, untreated Parkinson's disease. *Movement Disorders*, *24*(13), 1949–1954. https://doi.org/10.1002/mds.22680

Mille, E., Levin, J., Brendel, M., Zach, C., Barthel, H., Sabri, O., Bötzel, K., Bartenstein, P., Danek, A., & Rominger, A. (2017). Cerebral Glucose Metabolism and Dopaminergic

Function in Patients with Corticobasal Syndrome. *Journal of Neuroimaging*, *27*(2), 255–261. https://doi.org/10.1111/jon.12391

Minnerop, M., Lüders, E., Specht, K., Ruhlmann, J., Schimke, N., Thompson, P. M., Chou, Y. Y., Toga, A. W., Abele, M., Wüllner, U., & Klockgether, T. (2010). Callosal tissue loss in multiple system atrophy-A one-year follow-up study. *Movement Disorders*, *25*(15), 2613–2620. https://doi.org/10.1002/mds.23318

Minnerop, M., Specht, K., Ruhlmann, J., Schimke, N., Abele, M., Weyer, A., Wüllner, U., & Klockgether, T. (2007). Voxel-based morphometry and voxel-based relaxometry in multiple system atrophy-A comparison between clinical subtypes and correlations with clinical parameters. *NeuroImage*, *36*(4), 1086–1095. https://doi.org/10.1016/j.neuroimage.2007.04.028

Misch, M. R., Mitchell, S., Francis, P. L., Sherborn, K., Meradje, K., McNeely, A. A., Honjo, K., Zhao, J., Scott, C. J. M., Caldwell, C. B., Ehrlich, L., Shammi, P., MacIntosh, B. J., Bilbao, J. M., Lang, A. E., Black, S. E., & Masellis, M. (2014). Differentiating between visual hallucination-free dementia with Lewy bodies and corticobasal syndrome on the basis of neuropsychology and perfusion single-photon emission computed tomography. *Alzheimer's Research and Therapy*, *6*(9), 1–15. https://doi.org/10.1186/s13195-014-0071-4

Naduthota, R. M., Bharath, R. D., Jhunjhunwala, K., Yadav, R., Saini, J., Christopher, R., & Pal, P. K. (2017). Imaging biomarker correlates with oxidative stress in Parkinson's disease. *Neurology India*, *65*(2), 263–268. https://doi.org/10.4103/neuroindia.NI_981_15

Nagano-Saito, A., Washimi, Y., Arahata, Y., Kachi, T., Lerch, J. P., Evans, A. C., Dagher, A., & Ito, K. (2005). Cerebral atrophy and its relation to cognitive impairment in Parkinson disease. *Neurology*, *64*(2), 224–229. https://doi.org/10.1212/01.WNL.0000149510.41793.50

Nobili, F., Abbruzzese, G., Morbelli, S., Marchese, R., Girtler, N., Dessi, B., Brugnolo, A., Canepa, C., Drosos, G. C., Sambuceti, G., & Rodriguez, G. (2009). Amnestic mild cognitive impairment in Parkinson's disease: A brain perfusion SPECT study. *Movement Disorders*, *24*(3), 414–421. https://doi.org/10.1002/mds.22381

Padovani, A., Borroni, B., Brambati, S. M., Agosti, C., Broli, M., Alonso, R., Scifo, P., Bellelli, G., Alberici, A., Gasparotti, R., & Perani, D. (2006). Diffusion tensor imaging and voxel based morphometry study in early progressive supranuclear palsy. *Journal of Neurology, Neurosurgery and Psychiatry*, *77*(4), 457–463. https://doi.org/10.1136/jnnp.2005.075713

Pagonabarraga, J., Soriano-Mas, C., Llebaria, G., López-Solà, M., Pujol, J., & Kulisevsky, J. (2014). Neural correlates of minor hallucinations in non-demented patients with Parkinson's disease. *Parkinsonism and Related Disorders*, *20*(3), 290–296. https://doi.org/10.1016/j.parkreldis.2013.11.017

Pappatá, S., Santangelo, G., Aarsland, D., Vicidomini, C., Longo, K., Bronnick, K., Amboni, M., Erro, R., Vitale, C., Caprio, M. G., Pellecchia, M. T., Brunetti, A., De Michele, G.,

Salvatore, M., & Barone, P. (2011). Mild cognitive impairment in drug-naive patients with PD is associated with cerebral hypometabolism. *Neurology*, *77*(14), 1357–1362. https://doi.org/10.1212/WNL.0b013e3182315259

Pardini, M., Huey, E. D., Cavanagh, A. L., & Grafman, J. (2009). Olfactory function in corticobasal syndrome and frontotemporal dementia. *Archives of Neurology*, *66*(1), 92–96. https://doi.org/10.1001/archneurol.2008.521

Pardini, M., Huey, E. D., Spina, S., Kreisl, W. C., Morbelli, S., Wassermann, E. M., Nobili, F., Ghetti, B., & Grafman, J. (2019). FDG-PET patterns associated with underlying pathology in corticobasal syndrome. *Neurology*, *92*(10), E1121–E1135. https://doi.org/10.1212/WNL.0000000000007038

Park, H. K., Kim, J. S., Im, K. C., Oh, S. J., Kim, M. J., Lee, J. H., Chung, S. J., & Lee, M. C. (2009). Functional brain imaging in pure Akinesia with Gait freezing: [18F] FDG PET and [18F] FP-CIT PET analyses. *Movement Disorders*, *24*(2), 237–245. https://doi.org/10.1002/mds.22347

Pereira, J. B., Ibarretxe-Bilbao, N., Marti, M. J., Compta, Y., Junqué, C., Bargallo, N., & Tolosa, E. (2012). Assessment of cortical degeneration in patients with Parkinson's disease by voxel-based morphometry, cortical folding, and cortical thickness. *Human Brain Mapping*, *33*(11), 2521–2534. https://doi.org/10.1002/hbm.21378

Planetta, P. J., Kurani, A. S., Shukla, P., Prodoehl, J., Corcos, D. M., Comella, C. L., Mcfarland, N. R., Okun, M. S., & Vaillancourt, D. E. (2015). Distinct functional and macrostructural brain changes in Parkinson's disease and multiple system atrophy. *Human Brain Mapping*, *36*(3), 1165–1179. https://doi.org/10.1002/hbm.22694

Potgieser, A. R. E., Van Der Hoorn, A., Meppelink, A. M., Teune, L. K., Koerts, J., & De Jong, B. M. (2014). Anterior temporal atrophy and posterior progression in patients with parkinson's disease. *Neurodegenerative Diseases*, *14*(3), 125–132. https://doi.org/10.1159/000363245

Price, S., Paviour, D., Scahill, R., Stevens, J., Rossor, M., Lees, A., & Fox, N. (2004). Voxel-based morphometry detects patterns of atrophy that help differentiate progressive supranuclear palsy and Parkinson's disease. *NeuroImage*, *23*(2), 663–669. https://doi.org/10.1016/j.neuroimage.2004.06.013

Ramírez-Ruiz, B., Martí, M. J., Tolosa, E., Giménez, M., Bargalló, N., Valldeoriola, F., & Junqué, C. (2007). Cerebral atrophy in Parkinson's disease patients with visual hallucinations. *European Journal of Neurology*, *14*(7), 750–756. https://doi.org/10.1111/j.1468-1331.2007.01768.x

Rektorova, I., Biundo, R., Marecek, R., Weis, L., Aarsland, D., & Antonini, A. (2014). Grey matter changes in cognitively impaired Parkinson's disease patients. *PLoS ONE*, *9*(1). https://doi.org/10.1371/journal.pone.0085595

Ruppert, M. C., Greuel, A., Tahmasian, M., Schwartz, F., Stürmer, S., Maier, F., Hammes, J., Tittgemeyer, M., Timmermann, L., van Eimeren, T., Drzezga, A., & Eggers, C. (2020).

Network degeneration in Parkinson's disease: Multimodal imaging of nigro-striato-cortical dysfunction. *Brain*, *143*(3), 944–959. https://doi.org/10.1093/brain/awaa019

Sakurai, K., Imabayashi, E., Tokumaru, A. M., Hasebe, S., Murayama, S., Morimoto, S., Kanemaru, K., Takao, M., Shibamoto, Y., & Matsukawa, N. (2015). The feasibility of white matter volume reduction analysis using SPM8 plus DARTEL for the diagnosis of patients with clinically diagnosed corticobasal syndrome and Richardson's syndrome. *NeuroImage: Clinical*, *7*, 605–610. https://doi.org/10.1016/j.nicl.2014.02.009
Sakurai, K., Imabayashi, E., Tokumaru, A. M., Hasebe, S., Murayama, S., Morimoto, S., Kanemaru, K., Takao, M., Shibamoto, Y., & Matsukawa, N. (2015). The feasibility of white matter volume reduction analysis using SPM8 plus DARTEL for the diagnosis of patients with clinically diagnosed corticobasal syndrome and Richardson's syndrome. *NeuroImage: Clinical*, *7*, 605–610. https://doi.org/10.1016/j.nicl.2014.02.009
Sandhya, M., Saini, J., Pasha, S. A., Yadav, R., & Pal, P. K. (2014). A voxel based comparative analysis using magnetization transfer imaging and T1-weighted magnetic resonance imaging in progressive supranuclear palsy. *Annals of Indian Academy of Neurology*, *17*(2), 193–198. https://doi.org/10.4103/0972-2327.132626

Shen, B., Wei, S., Ge, J., Peng, S., Liu, F., Li, L., Guo, S., Wu, P., Zuo, C., Eidelberg, D., Wang, J., & Ma, Y. (2020). Reproducible metabolic topographies associated with multiple system atrophy: Network and regional analyses in Chinese and American patient cohorts. *NeuroImage: Clinical*, *28*(February), 102416. https://doi.org/10.1016/j.nicl.2020.102416

Shigemoto, Y., Matsuda, H., Kamiya, K., Maikusa, N., Nakata, Y., Ito, K., Ota, M., Matsunaga, N., & Sato, N. (2013). In vivo evaluation of gray and white matter volume loss in the parkinsonian variant of multiple system atrophy using SPM8 plus DARTEL for VBM. *NeuroImage: Clinical*, *2*(1), 491–496. https://doi.org/10.1016/j.nicl.2013.03.017

Song, I. U., Park, J. W., Chung, S. W., & Chung, Y. A. (2014). Brain SPECT can differentiate between essential tremor and early-stage tremor-dominant Parkinson's disease. *Journal of Clinical Neuroscience*, *21*(9), 1533–1537. https://doi.org/10.1016/j.jocn.2013.11.035

Specht, K., Minnerop, M., Abele, M., Reul, J., Wüllner, U., & Klockgether, T. (2003). In vivo voxel-based morphometry in multiple system atrophy of the cerebellar type. *Archives of Neurology*, *60*(10), 1431–1435. https://doi.org/10.1001/archneur.60.10.1431

Spotorno, N., Healey, M., McMillan, C. T., Rascovsky, K., Irwin, D. J., Clark, R., & Grossman, M. (2015). Processing ambiguity in a linguistic context: Decision-making difficulties in non-aphasic patients with behavioral variant frontotemporal degeneration. *Frontiers in Human Neuroscience*, *9*(OCTOBER), 1–8. https://doi.org/10.3389/fnhum.2015.00583

Srivastava, A., Sharma, R., Goyal, V., Chaudhary, S., Sood, S. K., & Kumaran, S. S. (2020). Saccadic Eye Movements in Young-Onset Parkinson's Disease - A BOLD fMRI Study. *Neuro-Ophthalmology*, *44*(2), 89–99. https://doi.org/10.1080/01658107.2019.1652656

Summerfield, C., Junqué, C., Tolosa, E., Salgado-Pineda, P., Gómez-Ansón, B., Martí, M. J., Pastor, P., Ramírez-Ruíz, B., & Mercader, J. (2005). Structural brain changes in parkinson disease with dementia: A voxel-based morphometry study. *Archives of Neurology, 62*(2), 281–285. https://doi.org/10.1001/archneur.62.2.281

Takahashi, R., Ishii, K., Kakigi, T., Yokoyama, K., Mori, E., & Murakami, T. (2011). Brain Alterations and Mini-Mental State Examination in Patients with Progressive Supranuclear Palsy: Voxel-Based Investigations Using ^{18}F-Fluorodeoxyglucose Positron Emission Tomography and Magnetic Resonance Imaging. *Dementia and Geriatric Cognitive Disorders Extra*, *1*(1), 381–392. https://doi.org/10.1159/000333368

Terada, T., Miyata, J., Obi, T., Kubota, M., Yoshizumi, M., & Murai, T. (2018). Reduced gray matter volume is correlated with frontal cognitive and behavioral impairments in Parkinson's disease. *Journal of the Neurological Sciences*, *390*(May), 231–238. https://doi.org/10.1016/j.jns.2018.05.005

Tessitore, A., Amboni, M., Cirillo, G., Corbo, D., Picillo, M., Russo, A., Vitale, C., Santangelo, G., Erro, R., Cirillo, M., Esposito, F., Barone, P., & Tedeschi, G. (2012). Regional gray matter atrophy in patients with Parkinson disease and freezing of gait. *American Journal of Neuroradiology*, *33*(9), 1804–1809. https://doi.org/10.3174/ajnr.A3066

Teune, L. K., Bartels, A. L., De Jong, B. M., Willemsen, A. T. M., Eshuis, S. A., De Vries, J. J., Van Oostrom, J. C. H., & Leenders, K. L. (2010). Typical cerebral metabolic patterns in neurodegenerative brain diseases. *Movement Disorders*, *25*(14), 2395–2404. https://doi.org/10.1002/mds.23291

Teune, L. K., Bartels, A. L., De Jong, B. M., Willemsen, A. T. M., Eshuis, S. A., De Vries, J. J., Van Oostrom, J. C. H., & Leenders, K. L. (2010). Typical cerebral metabolic patterns in neurodegenerative brain diseases. *Movement Disorders*, *25*(14), 2395–2404. https://doi.org/10.1002/mds.23291

Teune, L. K., Bartels, A. L., De Jong, B. M., Willemsen, A. T. M., Eshuis, S. A., De Vries, J. J., Van Oostrom, J. C. H., & Leenders, K. L. (2010). Typical cerebral metabolic patterns in neurodegenerative brain diseases. *Movement Disorders*, *25*(14), 2395–2404. https://doi.org/10.1002/mds.23291

Teune, L. K., Bartels, A. L., De Jong, B. M., Willemsen, A. T. M., Eshuis, S. A., De Vries, J. J., Van Oostrom, J. C. H., & Leenders, K. L. (2010). Typical cerebral metabolic patterns in neurodegenerative brain diseases. *Movement Disorders*, *25*(14), 2395–2404. https://doi.org/10.1002/mds.23291

Tir, M., Delmaire, C., le Thuc, V., Duhamel, A., Destée, A., Pruvo, J. P., & Defebvre, L. (2009). Motor-related circuit dysfunction in MSA-P: Usefulness of combined whole-brain imaging analysis. *Movement Disorders*, *24*(6), 863–870. https://doi.org/10.1002/mds.22463

Tir, M., Delmaire, C., le Thuc, V., Duhamel, A., Destée, A., Pruvo, J. P., & Defebvre, L. (2009). Motor-related circuit dysfunction in MSA-P: Usefulness of combined whole-

brain imaging analysis. *Movement Disorders*, *24*(6), 863–870. https://doi.org/10.1002/mds.22463

Tzarouchi, L. C., Astrakas, L. G., Konitsiotis, S., Tsouli, S., Margariti, P., Zikou, A., & Argyropoulou, M. I. (2010). Voxel-based morphometry and voxel-based relaxometry in parkinsonian variant of multiple system atrophy. *Journal of Neuroimaging*, *20*(3), 260–266. https://doi.org/10.1111/j.1552-6569.2008.00343.x

Van Laere, K., Santens, P., Bosman, T., De Reuck, J., Mortelmans, L., & Dierckx, R. (2004). Statistical parametric mapping of 99mTc-ECD SPECT in idiopathic Parkinson’s disease and multiple system atrophy with predominant parkinsonian features: Correlation with clinical parameters. *Journal of Nuclear Medicine*, *45*(6), 933–942.

Van Laere, K., Santens, P., Bosman, T., De Reuck, J., Mortelmans, L., & Dierckx, R. (2004). Statistical parametric mapping of 99mTc-ECD SPECT in idiopathic Parkinson’s disease and multiple system atrophy with predominant parkinsonian features: Correlation with clinical parameters. *Journal of Nuclear Medicine*, *45*(6), 933–942.

Varrone, A., Pagani, M., Salvatore, E., Salmaso, D., Sansone, V., Amboni, M., Nobili, F., De Michele, G., Filla, A., Barone, P., Pappatà, S., & Salvatore, M. (2007). Identification by [99mTc]ECD SPECT of anterior cingulate hypoperfusion in progressive supranuclear palsy, in comparison with Parkinson’s disease. *European Journal of Nuclear Medicine and Molecular Imaging*, *34*(7), 1071–1081. https://doi.org/10.1007/s00259-006-0344-7

Wang, G., Wang, J., Zhan, J., Nie, B., Li, P., Fan, L., Zhu, H., Feng, T., & Shan, B. (2015). Quantitative assessment of cerebral gray matter density change in progressive supranuclear palsy using voxel based morphometry analysis and cerebral MR T1-weighted FLAIR imaging. *Journal of the Neurological Sciences*, *359*(1–2), 367–372. https://doi.org/10.1016/j.jns.2015.11.007

Wang, X., Zhang, J., Yuan, Y., Li, T., Zhang, L., Ding, J., Jiang, S., Li, J., Zhu, L., & Zhang, K. (2017). Cerebral metabolic change in Parkinson’s disease patients with anxiety: A FDG-PET study. *Neuroscience Letters*, *653*, 202–207. https://doi.org/10.1016/j.neulet.2017.05.062

Whitwell, J. L., Duffy, J. R., Strand, E. A., Machulda, M. M., Senjem, M. L., Gunter, J. L., Kantarci, K., Eggers, S. D., Jack, C. R., & Josephs, K. A. (2013). Neuroimaging comparison of primary progressive apraxia of speech and progressive supranuclear palsy. *European Journal of Neurology*, *20*(4), 629–637. https://doi.org/10.1111/ene.12004

Whitwell, J. L., Jack, C. R., Parisi, J. E., Knopman, D. S., Boeve, B. F., Petersen, R. C., Dickson, D. W., & Josephs, K. A. (2011). Imaging signatures of molecular pathology in behavioral variant frontotemporal dementia. *J Mol Neurosci*, *45*(3), 372–378. https://doi.org/10.1007/s12031-011-9533-3.Imaging

Wu, Y. N., Chen, M. H., Chiang, P. L., Lu, C. H., Chen, H. L., Yu, C. C., Chen, Y. S., Chang, Y. Y., & Lin, W. C. (2020). Associations between brain structural damage and core muscle loss in patients with parkinson’s disease. *Journal of Clinical Medicine*, *9*(1), 1–12. https://doi.org/10.3390/jcm9010239

Xuan, M., Guan, X., Huang, P., Shen, Z., Gu, Q., Yu, X., Xu, X., Luo, W., & Zhang, M. (2019). Different patterns of gray matter density in early- and middle-late-onset Parkinson's disease: a voxel-based morphometry study. *Brain Imaging and Behavior*, *13*(1), 172–179. https://doi.org/10.1007/s11682-017-9745-4

Yang, Y. J., Ge, J. J., Liu, F. T., Liu, Z. Y., Zhao, J., Wu, J. J., Ma, Y., Zuo, C. T., & Wang, J. (2019). Preserved caudate function in young-onset patients with Parkinson's disease: a dual-tracer PET imaging study. *Therapeutic Advances in Neurological Disorders*, *12*, 1–11. https://doi.org/10.1177/1756286419851400

Yu, C. C., Chen, H. L., Chen, M. H., Lu, C. H., Tsai, N. W., Huang, C. C., Chang, Y. Y., Li, S. H., Chen, Y. S., Chiang, P. L., & Lin, W. C. (2020). Vascular Inflammation Is a Risk Factor Associated with Brain Atrophy and Disease Severity in Parkinson's Disease: A Case-Control Study. *Oxidative Medicine and Cellular Longevity*, *2020*. https://doi.org/10.1155/2020/2591248

Zamboni, G., Grafman, J., Krueger, F., Knutson, K. M., & Huey, E. D. (2010). Anosognosia for behavioral disturbances in frontotemporal dementia and corticobasal syndrome: A voxel-based morphometry study. *Dementia and Geriatric Cognitive Disorders*, *29*(1), 88–96. https://doi.org/10.1159/000255141

Zhang, J., Zhang, Y. T., Hu, W. D., Li, L., Liu, G. Y., & Bai, Y. P. (2015). Gray matter atrophy in patients with Parkinson's disease and those with mild cognitive impairment: A voxel-based morphometry study. *International Journal of Clinical and Experimental Medicine*, *8*(9), 15383–15392.

Supplementary Table 3

Consistent Regions of Abnormality in Parkinsonian Disorders with Citations.

Contrast		Region	x	y	z	Volume (mm^3)	ALE Value	Convergence *n* (%)	Contributing Studies
PET ***PD < HC*** *Number of experiments in analysis = 17*	1	L. Mid. Temporal G.	-44.7	-64.4	35.8	2088	0.0251	8 (47%)	(Chu et al., 2019; Chung et al., 2016; Gasca-Salas et al., 2016; Hosey et al., 2005; Hosokai et al., 2009; Huang et al., 2013; Le Jeune et al., 2010; Lyoo et al., 2010)
	2	L. Caudate	-14.3	13.1	6.6	1000	0.0251	5 (29.4%)	(Berding et al., 2001; Berti et al., 2011; Chung et al., 2016; Juh et al., 2004; X. Wang et al., 2017)
	3	R. Inferior Frontal G.	57.4	16.4	23.6	680	0.0253	3(17.6%)	(Berti et al., 2011; Hosokai et al., 2009; Teune et al., 2010a)
	4	R. Middle Frontal G.	34	22	42.8	664	0.0223	3(17.6%)	(Chung et al., 2016; Gasca-Salas et al., 2016; Lyoo et al., 2010)
MRI ***PSP < HC***	1	Bilat. Thalamus/Red nucleus	2.2	-14.1	-0.9	11072	0.0488	14 (87.5%)	(Agosta et al., 2010; Boxer et al., 2006; Brenneis et al., 2004; Cordato et al., 2005; Ghosh et al., 2012; Lagarde et al., 2013, 2015; Lehéricy et al., 2010; Padovani et al., 2006; Price et al., 2004; Sakurai et al., 2015; Takahashi et al., 2011; G. Wang et al., 2015; Whitwell et al., 2013)
Number of experiments in analysis = 16	2	R. Insula	43.5	17.2	4.1	1040	0.023	4 (25%)	(Boxer et al., 2006; Ghosh et al., 2012; Padovani et al., 2006; G. Wang et al., 2015)
	3	L. Caudate	-10.7	6.2	12.9	952	0.0257	6 (37.5%)	(Agosta et al., 2010; Boxer et al., 2006; Cordato et al., 2005; Lehéricy et al., 2010; G. Wang et al., 2015; Whitwell et al., 2013)
	4	L. Brainstem	-7.4	-34.9	-13.5	816	0.0231	4 (25%)	(Agosta et al., 2010; Ghosh et al., 2012; Lehéricy et al., 2010; G. Wang et al., 2015)
	5	L. Insula	-37	16.9	3.5	808	0.0218	4 (25%)	(Boxer et al., 2006; Brenneis et al., 2004; Takahashi et al., 2011; Whitwell et al., 2013)
MRI ***MSA < HC***	1	Brainstem	1.2	-33.8	-18.9	896	0.0256	4 (30.8%)	(Brenneis et al., 2006; Minnerop et al., 2007, 2010; Shigemoto et al., 2013)
Number of experiments in analysis = 13	2	L. Putamen	-22.7	11.6	-6.4	776	0.0209	4 (30.8%)	(Cao et al., 2021; Minnerop et al., 2010; Shigemoto et al., 2013; Tzarouchi et al., 2010)

	3	Brainstem	-12	-35.3	-32	640	0.0162	5 (38.5%)	(Brenneis et al., 2006; Minnerop et al., 2007; Shigemoto et al., 2013; Specht et al., 2003; Tzarouchi et al., 2010)
MRI Parkinsonian disorders < HC *Number of experiments in analysis* = 83	1	R. Thalamus[*4/4]	5.4	-11.9	13.5	2744	0.0498	12 (14.3%)	(Agosta et al., 2010; Burton et al., 2004; Cordato et al., 2005; Huey et al., 2009; Lagarde et al., 2013, 2015; Minnerop et al., 2010; Padovani et al., 2006; Pardini et al., 2009; Tzarouchi et al., 2010; G. Wang et al., 2015; Whitwell et al., 2013)
	2	L. Caudate[*3/4]	-26.3	13.9	6.3	2312	0.0385	16 (19%)	(Agosta et al., 2010; Boxer et al., 2006; Brenneis et al., 2004; Ghosh et al., 2012; Lagarde et al., 2013; Lehéricy et al., 2010; R. Li et al., 2022; X. Li et al., 2017; Minnerop et al., 2007; Naduthota et al., 2017; Potgieser et al., 2014; Price et al., 2004; Sakurai et al., 2015; Shigemoto et al., 2013; Takahashi et al., 2011; Tzarouchi et al., 2010; G. Wang et al., 2015; Whitwell et al., 2013)
	3	Bilat. Midbrain/Red nucleus[*2/4]	1.6	-17.5	-9	1432	0.0538	12 (14.3%)	(Agosta et al., 2010; Boxer et al., 2006; Brenneis et al., 2004, 2006; Cheng et al., 2020; Cordato et al., 2005; Guimarães et al., 2017; Minnerop et al., 2007; Planetta et al., 2015; Shigemoto et al., 2013; Summerfield et al., 2005; Takahashi et al., 2011; Tzarouchi et al., 2010; G. Wang et al., 2015; Whitwell et al., 2013; Xuan et al., 2019)
	4	L. Amygdala[*3/4]	-19.7	-8.4	-11.7	1016	0.0305	10 (11.9%)	(Agosta et al., 2010; Ghosh et al., 2012; R. Li et al., 2022; X. Li et al., 2017; Naduthota et al., 2017; Potgieser et al., 2014; Price et al., 2004; Sakurai et al., 2015; Shigemoto et al., 2013; G. Wang et al., 2015)
	5	R. Parahippocampal G., Amygdala[*3/4]	19	-12.4	-14.4	1008	0.0349	7 (8.3%)	(Agosta et al., 2010; Chang et al., 2009; Lehéricy et al., 2010; Minnerop et al., 2007; Sakurai et al., 2015; Srivastava et al., 2020; Takahashi et al., 2011)
	6	L. Brainstem[*2/4]	-3.2	-33.4	-16	856	0.0302	6 (7.1%)	(Agosta et al., 2010; Lehéricy et al., 2010; Minnerop et al., 2007, 2010; Shigemoto et al., 2013; G. Wang et al., 2015)
PET Parkinsonian disorders < HC	1	L. Lateral occipital cortex[*3/4]	-45.5	-64.2	34.3	2344	0.0319	10 (30.3%)	(Chu et al., 2019; Chung et al., 2016; Gasca-Salas et al., 2016; Grimaldi et al., 2019; Hosey et al., 2005; Hosokai et al., 2009; Le Jeune et al., 2010; Lyoo et al., 2008, 2010; Park et al., 2009)
Number of experiments in analysis = 34	2	L. Caudate[*4/4]	-14.9	11.9	7.5	2056	0.0414	11 (33.3%)	(Berti et al., 2011; Chung et al., 2016; Gasca-Salas et al., 2016; Ge et al., 2018; Juh et al.,

									2004; Lyoo et al., 2008; Pardini et al., 2019; Shen et al., 2020; X. Wang et al., 2017)
	3	R. Caudate$^{*4/4}$	16.2	13.7	4.9	1840	0.0345	10 (30.3%)	(Berding et al., 2001; Berti et al., 2011; Chu et al., 2019; Hosaka et al., 2002; Lyoo et al., 2008; Pardini et al., 2019; Shen et al., 2020; Teune et al., 2010b; X. Wang et al., 2017)
	4	R. Inferior frontal G.$^{*4/4}$	57.2	15	23.3	1296	0.0403	8 (24.2%)	(Berti et al., 2011; Hosaka et al., 2002; Hosokai et al., 2009; Lyoo et al., 2008; Teune et al., 2010b)
	5	R. Middle frontal G.$^{*2/4}$	34	20.3	45.9	848	0.0228	5 (15.2%)	(Chung et al., 2016; Gasca-Salas et al., 2016; Grimaldi et al., 2019; Hosokai et al., 2009; Lyoo et al., 2010)
	6	R. Lateral Occipital corext$^{*3/4}$	42	-60	47.3	752	0.0212	4 (12.1%)	(Berding et al., 2001; Gasca-Salas et al., 2016; Teune et al., 2010b)
Parkinsonian disorders > HC	1	R. Middle Temporal G.$^{*4/4}$	39.5	-31.8	-2.8	1200	0.0159	5 (45.5%)	(Huang et al., 2013; Teune et al., 2010b)
Number of experiments in analysis = 11	2	L. Insular cortex$^{*3/4}$	-32.6	-18.5	21.7	792	0.015	4 (36.4%)	(Ge et al., 2018; Hosey et al., 2005; Teune et al., 2010b)
	3	L. Inferior Occipital G.$^{*3/4}$	-40.3	-81.6	1.6	648	0.0178	3 (27.3%)	(Teune et al., 2010b)

Note.
All clusters reported were significant at $p < .05$ family-wise error corrected. Centre of gravity provided in MNI x,y,z coordinates. Some studies included multiple patient groups and therefore performed multiple experiments. Some studies contributed coordinates from multiple patient populations, these are as follows: Parkinsonian disorders < HC PET; Cluster 2: Juh et al., 2004 contributed coordinates for both patients with PSP and PD. Parkinsonian disorders < HC PET; Cluster 3: Teune et al., 2010 contributed coordinates from Patients with CBS and PSP. Parkinsonian disorders < HC PET; Cluster 4: Teune et al., 2010 contributed coordinates from patients with PD, MSA and PSP; Hosaka et al., 2002 contributed coordinates from patients with CBS and PSP. Cluster 6: Teune et al., 2010 contributed coordinates from patients with MSA and CBS. Parkinsonian disorders > HC PET; Cluster 1: Teune et al., 2010 contributed coordinates from PD, PSP, CBS and MSA. Parkinsonian disorders > HC PET; Cluster 2: Teune et al., contributed coordinates from patients with PSP and MSA. Parkinsonian disorders > HC PET; Cluster 3: Teune et al., 2010 contributed coordinates from patients with PD, PSP, CBS and MSA. Convergence n = number of experiments contributing to clusters. R. = Right; L. = Left; G. = Gyrus; Bilat. = Bilateral; Mid. = Middle; Inf. = Inferior; PD = Parkinson's disease; PSP = Progressive supranuclear palsy; MSA = Multiple system atrophy; HC = Healthy controls; MNI = Montreal Neurological Institute. *denotes the number of disorders in the parkinsonism analyses that contributed to the significant result.

Supplementary References (Supplementary Table 3)

Agosta, F., Kostić, V. S., Galantucci, S., Mesaroš, Š., Svetel, M., Pagani, E., Stefanova, E., & Filippi, M. (2010). The in vivo distribution of brain tissue loss in Richardson's syndrome and PSP-parkinsonism: A VBM-DARTEL study. *European Journal of Neuroscience*, *32*(4), 640–647. https://doi.org/10.1111/j.1460-9568.2010.07304.x

Berding, G., Odin, P., Brooks, D. J., Nikkhah, G., Matthies, C., Peschel, T., Shing, M., Kolbe, H., Hoff, J. van den, Fricke, H., Dengler, R., Samii, M., & Knapp, W. H. (2001). Resting Regional Cerebral Glucose Metabolism in Advanced Parkinson's Disease Studied in the Offand On Conditions with [18F]FDG-PET. *Movement Disorders*, *16*(6), 1007–1013. https://doi.org/10.1002/mds.1221

Berti, V., Polito, C., Borghammer, P., Ramat, S., Mosconi4, L., E. Vanzi1, M. T. De, Cristofaro1, M., De Leon, S., Sorbi, & Pupi, A. (2011). Alternative normalization methods demonstrate widespread cortical hypometabolism in untreated de novo Parkinson's disease. *J Nucl Med Mol Imaging*, *23*(1), 1–7. https://www.ncbi.nlm.nih.gov/pmc/articles/PMC3624763/pdf/nihms412728.pdf

Boxer, A. L., Geschwind, M. D., Belfor, N., Gorno-Tempini, M. L., Schauer, G. F., Miller, B. L., Weiner, M. W., & Rosen, H. J. (2006). Patterns of brain atrophy that differentiate corticobasal degeneration syndrome from progressive supranuclear palsy. *Archives of Neurology*, *63*(1), 81–86. https://doi.org/10.1001/archneur.63.1.81

Brenneis, C., Boesch, S. M., Egger, K. E., Seppi, K., Scherfler, C., Schocke, M., Wenning, G. K., & Poewe, W. (2006). Cortical atrophy in the cerebellar variant of multiple system atrophy: A voxel-based morphometry study. *Movement Disorders*, *21*(2), 159–165. https://doi.org/10.1002/mds.20656

Brenneis, C., Seppi, K., Schocke, M., Benke, T., Wenning, G. K., & Poewe, W. (2004). Voxel based morphometry reveals a distinct pattern of frontal atrophy in progressive supranuclear palsy. *Journal of Neurology, Neurosurgery and Psychiatry*, *75*(2), 246–249. https://doi.org/10.1136/jnnp.2003.015297

Burton, E. J., McKeith, I. G., Burn, D. J., Williams, E. D., & O'Brien, J. T. (2004). Cerebral atrophy in Parkinson's disease with and without dementia: A comparison with Alzheimer's disease, dementia with Lewy bodies and controls. *Brain*, *127*(4), 791–800. https://doi.org/10.1093/brain/awh088

Cao, C., Wang, Q., Yu, H., Yang, H., Li, Y., Guo, M., Huo, H., & Fan, G. (2021). Morphological Changes in Cortical and Subcortical Structures in Multiple System Atrophy Patients With Mild Cognitive Impairment. *Frontiers in Human Neuroscience*, *15*(March), 1–8. https://doi.org/10.3389/fnhum.2021.649051

Chang, C. C., Chang, Y. Y., Chang, W. N., Lee, Y. C., Wang, Y. L., Lui, C. C., Huang, C. W., & Liu, W. L. (2009). Cognitive deficits in multiple system atrophy correlate with frontal atrophy and disease duration. *European Journal of Neurology*, *16*(10), 1144–1150. https://doi.org/10.1111/j.1468-1331.2009.02661.x

Cheng, L., Wu, X., Guo, R., Wang, Y., Wang, W., He, P., Lin, H., & Shen, J. (2020). Discriminative pattern of reduced cerebral blood flow in Parkinson's disease and Parkinsonism-Plus syndrome: An ASL-MRI study. *BMC Medical Imaging*, *20*(1), 1–9. https://doi.org/10.1186/s12880-020-00479-y

Chu, J. S., Liu, T. H., Wang, K. L., Han, C. L., Liu, Y. P., Michitomo, S., Zhang, J. G., Fang, T., & Meng, F. G. (2019). The metabolic activity of caudate and prefrontal cortex negatively correlates with the severity of idiopathic Parkinson's disease. *Aging and Disease*, *10*(4), 847–853. https://doi.org/10.14336/AD.2018.0814

Chung, E. J., Han, Y. H., Mun, C. W., Bae, S. K., Lee, S. M., Jeong, H. W., & Kim, S. J. (2016). Hypometabolism based on a cutoff point on the mini-mental state examination in Parkinson's disease. *Neurology Asia*, *21*(3), 247–253.

Cordato, N. J., Duggins, A. J., Halliday, G. M., Morris, J. G. L., & Pantelis, C. (2005). Clinical deficits correlate with regional cerebral atrophy in progressive supranuclear palsy. *Brain*, *128*(6), 1259–1266. https://doi.org/10.1093/brain/awh508

Gasca-Salas, C., Clavero, P., García-García, D., Obeso, J. A., & Rodríguez-Oroz, M. C. (2016). Significance of visual hallucinations and cerebral hypometabolism in the risk of dementia in Parkinson's disease patients with mild cognitive impairment. *Human Brain Mapping*, *37*(3), 968–977. https://doi.org/10.1002/hbm.23080

Ge, J., Wu, J., Peng, S., Wu, P., Wang, J., Zhang, H., Guan, Y., Eidelberg, D., Zuo, C., & Ma, Y. (2018). Reproducible network and regional topographies of abnormal glucose metabolism associated with progressive supranuclear palsy: Multivariate and univariate analyses in American and Chinese patient cohorts. *Human Brain Mapping*, *39*(7), 2842–2858. https://doi.org/10.1002/hbm.24044

Ghosh, B. C. P., Calder, A. J., Peers, P. V., Lawrence, A. D., Acosta-Cabronero, J., Pereira, J. M., Hodges, J. R., & Rowe, J. B. (2012). Social cognitive deficits and their neural correlates in progressive supranuclear palsy. *Brain*, *135*(7), 2089–2102. https://doi.org/10.1093/brain/aws128

Grimaldi, S., Boucekine, M., Witjas, T., Fluchère, F., Renaud, M., Azulay, J. P., Guedj, E., & Eusebio, A. (2019). Multiple System Atrophy: Phenotypic spectrum approach coupled with brain 18-FDG PET. *Parkinsonism and Related Disorders*, *67*(September), 3–9. https://doi.org/10.1016/j.parkreldis.2019.09.005

Guimarães, R. P., Santos, M. C. A., Dagher, A., Campos, L. S., Azevedo, P., Piovesana, L. G., De Campos, B. M., Larcher, K., Zeighami, Y., Amato-Filho, A. C. S., Cendes, F., & Frota D'Abreu, A. C. (2017). Pattern of reduced functional connectivity and structural abnormalities in Parkinson's disease: An exploratory study. *Frontiers in Neurology, 7*. https://doi.org/10.3389/fneur.2016.00243

Hosaka, K., Ishii, K., Sakamoto, S., Mori, T., Sasaki, M., Hirono, N., & Mori, E. (2002). Voxel-based comparison of regional cerebral glucose metabolism between PSP and corticobasal degeneration. *Journal of the Neurological Sciences*, *199*(1–2), 67–71. https://doi.org/10.1016/S0022-510X(02)00102-8

Hosey, L. A., Thompson, J. L. W., Verhagen Metman, L., Van Den Munckhof, P., & Braun, A. R. (2005). Temporal dynamics of cortical and subcortical responses to apomorphine in Parkinson disease: An H215O PET study. *Clinical Neuropharmacology*, *28*(1), 18–27. https://doi.org/10.1097/01.wnf.0000154220.30263.0e

Hosokai, Y., Nishio, Y., Hirayama, K., Takeda, A., Ishioka, T., Sawada, Y., Suzuki, K., Itoyama, Y., Takahashi, S., Fukuda, H., & Mori, E. (2009). Distinct patterns of regional cerebral glucose metabolism in Parkinson's disease with and without mild cognitive impairment. *Movement Disorders*, *24*(6), 854–862. https://doi.org/10.1002/mds.22444

Huang, C., Ravdin, L. D., Nirenberg, M. J., Piboolnurak, P., Severt, L., Maniscalco, J. S., Solnes, L., Dorfman, B. J., & Henchcliffe, C. (2013). Neuroimaging markers of motor and nonmotor features of parkinson's disease: An [18F]fluorodeoxyglucose positron emission computed tomography study. *Dementia and Geriatric Cognitive Disorders*, *35*(3–4), 183–196. https://doi.org/10.1159/000345987

Huey, E. D., Pardini, M., Cavanagh, A., Wassermann, E. M., Kapogiannis, D., Spina, S., Ghetti, B., & Grafman, J. (2009). Association of ideomotor apraxia with frontal gray matter volume loss in corticobasal syndrome. *Archives of Neurology*, *66*(10), 1274–1280. https://doi.org/10.1001/archneurol.2009.218

Juh, R., Kim, J., Moon, D., Choe, B., & Suh, T. (2004). Different metabolic patterns analysis of Parkinsonism on the 18F-FDG PET. *European Journal of Radiology*, *51*(3), 223–233. https://doi.org/10.1016/S0720-048X(03)00214-6

Lagarde, J., Valabrégue, R., Corvol, J. C., Garcin, B., Volle, E., Le Ber, I., Vidailhet, M., Dubois, B., & Levy, R. (2015). Why do patients with neurodegenerative frontal syndrome fail to answer: "In what way are an orange and a banana alike?" *Brain*, *138*(2), 456–471. https://doi.org/10.1093/brain/awu359

Lagarde, J., Valabrègue, R., Corvol, J. C., Pineau, F., Le Ber, I., Vidailhet, M., Dubois, B., & Levy, R. (2013). Are frontal cognitive and atrophy patterns different in PSP and bvFTD? A comparative neuropsychological and VBM study. *PLoS ONE*, *8*(11), 1–10. https://doi.org/10.1371/journal.pone.0080353

Le Jeune, F., Péron, J., Grandjean, D., Drapier, S., Haegelen, C., Garin, E., Millet, B., & Vérin, M. (2010). Subthalamic nucleus stimulation affects limbic and associative circuits: A PET study. *European Journal of Nuclear Medicine and Molecular Imaging*, *37*(8), 1512–1520. https://doi.org/10.1007/s00259-010-1436-y

Lehéricy, S., Hartmann, A., Lannuzel, A., Galanaud, D., Delmaire, C., Bienaimée, M. J., Jodoin, N., Roze, E., Gaymard, B., & Vidailhet, M. (2010). Magnetic resonance imaging lesion pattern in Guadeloupean parkinsonism is distinct from progressive supranuclear palsy. *Brain*, *133*(8), 2410–2425. https://doi.org/10.1093/brain/awq162

Li, R., Zou, T., Wang, X., Wang, H., Hu, X., Xie, F., Meng, L., & Chen, H. (2022). Basal ganglia atrophy–associated causal structural network degeneration in Parkinson's disease. *Human Brain Mapping*, *43*(3), 1145–1156. https://doi.org/10.1002/hbm.25715

Li, X., Xing, Y., Schwarz, S. T., & Auer, D. P. (2017). Limbic grey matter changes in early Parkinson's disease. *Human Brain Mapping*, *38*(7), 3566–3578. https://doi.org/10.1002/hbm.23610

Lyoo, C. H., Jeong, Y., Ryu, Y. H., Lee, S. Y., Song, T. J., Lee, J. H., Rinne, J. O., & Lee, M. S. (2008). Effects of disease duration on the clinical features and brain glucose metabolism in patients with mixed type multiple system atrophy. *Brain*, *131*(2), 438–446. https://doi.org/10.1093/brain/awm328

Lyoo, C. H., Jeong, Y., Ryu, Y. H., Rinne, J. O., & Lee, M. S. (2010). Cerebral glucose metabolism of Parkinson's disease patients with mild cognitive impairment. *European Neurology*, *64*(2), 65–73. https://doi.org/10.1159/000315036

Minnerop, M., Lüders, E., Specht, K., Ruhlmann, J., Schimke, N., Thompson, P. M., Chou, Y. Y., Toga, A. W., Abele, M., Wüllner, U., & Klockgether, T. (2010). Callosal tissue loss in multiple system atrophy-A one-year follow-up study. *Movement Disorders*, *25*(15), 2613–2620. https://doi.org/10.1002/mds.23318

Minnerop, M., Specht, K., Ruhlmann, J., Schimke, N., Abele, M., Weyer, A., Wüllner, U., & Klockgether, T. (2007). Voxel-based morphometry and voxel-based relaxometry in multiple system atrophy-A comparison between clinical subtypes and correlations with clinical parameters. *NeuroImage*, *36*(4), 1086–1095. https://doi.org/10.1016/j.neuroimage.2007.04.028

Naduthota, R. M., Bharath, R. D., Jhunjhunwala, K., Yadav, R., Saini, J., Christopher, R., & Pal, P. K. (2017). Imaging biomarker correlates with oxidative stress in Parkinson's disease. *Neurology India*, *65*(2), 263–268. https://doi.org/10.4103/neuroindia.NI_981_15

Padovani, A., Borroni, B., Brambati, S. M., Agosti, C., Broli, M., Alonso, R., Scifo, P., Bellelli, G., Alberici, A., Gasparotti, R., & Perani, D. (2006). Diffusion tensor imaging and voxel based morphometry study in early progressive supranuclear palsy. *Journal of Neurology, Neurosurgery and Psychiatry*, *77*(4), 457–463. https://doi.org/10.1136/jnnp.2005.075713

Pardini, M., Huey, E. D., Cavanagh, A. L., & Grafman, J. (2009). Olfactory function in corticobasal syndrome and frontotemporal dementia. *Archives of Neurology*, *66*(1), 92–96. https://doi.org/10.1001/archneurol.2008.521

Pardini, M., Huey, E. D., Spina, S., Kreisl, W. C., Morbelli, S., Wassermann, E. M., Nobili, F., Ghetti, B., & Grafman, J. (2019). FDG-PET patterns associated with underlying pathology in corticobasal syndrome. *Neurology*, *92*(10), E1121–E1135. https://doi.org/10.1212/WNL.0000000000007038

Park, H. K., Kim, J. S., Im, K. C., Oh, S. J., Kim, M. J., Lee, J. H., Chung, S. J., & Lee, M. C. (2009). Functional brain imaging in pure Akinesia with Gait freezing: [18F] FDG PET and [18F] FP-CIT PET analyses. *Movement Disorders*, *24*(2), 237–245. https://doi.org/10.1002/mds.22347

Planetta, P. J., Kurani, A. S., Shukla, P., Prodoehl, J., Corcos, D. M., Comella, C. L., Mcfarland, N. R., Okun, M. S., & Vaillancourt, D. E. (2015). Distinct functional and macrostructural brain changes in Parkinson's disease and multiple system atrophy. *Human Brain Mapping*, *36*(3), 1165–1179. https://doi.org/10.1002/hbm.22694

Potgieser, A. R. E., Van Der Hoorn, A., Meppelink, A. M., Teune, L. K., Koerts, J., & De Jong, B. M. (2014). Anterior temporal atrophy and posterior progression in patients with parkinson's disease. *Neurodegenerative Diseases*, *14*(3), 125–132. https://doi.org/10.1159/000363245

Price, S., Paviour, D., Scahill, R., Stevens, J., Rossor, M., Lees, A., & Fox, N. (2004). Voxel-based morphometry detects patterns of atrophy that help differentiate progressive supranuclear palsy and Parkinson's disease. *NeuroImage*, *23*(2), 663–669. https://doi.org/10.1016/j.neuroimage.2004.06.013

Sakurai, K., Imabayashi, E., Tokumaru, A. M., Hasebe, S., Murayama, S., Morimoto, S., Kanemaru, K., Takao, M., Shibamoto, Y., & Matsukawa, N. (2015). The feasibility of white matter volume reduction analysis using SPM8 plus DARTEL for the diagnosis of patients with clinically diagnosed corticobasal syndrome and Richardson's syndrome. *NeuroImage: Clinical*, *7*, 605–610. https://doi.org/10.1016/j.nicl.2014.02.009

Shen, B., Wei, S., Ge, J., Peng, S., Liu, F., Li, L., Guo, S., Wu, P., Zuo, C., Eidelberg, D., Wang, J., & Ma, Y. (2020). Reproducible metabolic topographies associated with multiple system atrophy: Network and regional analyses in Chinese and American patient cohorts. *NeuroImage: Clinical*, *28*(February), 102416. https://doi.org/10.1016/j.nicl.2020.102416

Shigemoto, Y., Matsuda, H., Kamiya, K., Maikusa, N., Nakata, Y., Ito, K., Ota, M., Matsunaga, N., & Sato, N. (2013). In vivo evaluation of gray and white matter volume loss in the parkinsonian variant of multiple system atrophy using SPM8 plus DARTEL for VBM. *NeuroImage: Clinical*, *2*(1), 491–496. https://doi.org/10.1016/j.nicl.2013.03.017

Specht, K., Minnerop, M., Abele, M., Reul, J., Wüllner, U., & Klockgether, T. (2003). In vivo voxel-based morphometry in multiple system atrophy of the cerebellar type. *Archives of Neurology*, *60*(10), 1431–1435. https://doi.org/10.1001/archneur.60.10.1431

Srivastava, A., Sharma, R., Goyal, V., Chaudhary, S., Sood, S. K., & Kumaran, S. S. (2020). Saccadic Eye Movements in Young-Onset Parkinson's Disease - A BOLD fMRI Study. *Neuro-Ophthalmology*, *44*(2), 89–99. https://doi.org/10.1080/01658107.2019.1652656

Summerfield, C., Junqué, C., Tolosa, E., Salgado-Pineda, P., Gómez-Ansón, B., Martí, M. J., Pastor, P., Ramírez-Ruíz, B., & Mercader, J. (2005). Structural brain changes in parkinson disease with dementia: A voxel-based morphometry study. *Archives of Neurology*, *62*(2), 281–285. https://doi.org/10.1001/archneur.62.2.281

Takahashi, R., Ishii, K., Kakigi, T., Yokoyama, K., Mori, E., & Murakami, T. (2011). Brain Alterations and Mini-Mental State Examination in Patients with Progressive Supranuclear Palsy: Voxel-Based Investigations Using ^{18}F-Fluorodeoxyglucose Positron Emission Tomography and Magnetic Resonance Imaging. *Dementia and Geriatric Cognitive Disorders Extra*, *1*(1), 381–392. https://doi.org/10.1159/000333368

Teune, L. K., Bartels, A. L., De Jong, B. M., Willemsen, A. T. M., Eshuis, S. A., De Vries, J. J., Van Oostrom, J. C. H., & Leenders, K. L. (2010a). Typical cerebral metabolic patterns in neurodegenerative brain diseases. *Movement Disorders*, *25*(14), 2395–2404. https://doi.org/10.1002/mds.23291

Teune, L. K., Bartels, A. L., De Jong, B. M., Willemsen, A. T. M., Eshuis, S. A., De Vries, J. J., Van Oostrom, J. C. H., & Leenders, K. L. (2010b). Typical cerebral metabolic patterns in neurodegenerative brain diseases. *Movement Disorders*, *25*(14), 2395–2404. https://doi.org/10.1002/mds.23291

Tzarouchi, L. C., Astrakas, L. G., Konitsiotis, S., Tsouli, S., Margariti, P., Zikou, A., & Argyropoulou, M. I. (2010). Voxel-based morphometry and voxel-based relaxometry in parkinsonian variant of multiple system atrophy. *Journal of Neuroimaging*, *20*(3), 260–266. https://doi.org/10.1111/j.1552-6569.2008.00343.x

Wang, G., Wang, J., Zhan, J., Nie, B., Li, P., Fan, L., Zhu, H., Feng, T., & Shan, B. (2015). Quantitative assessment of cerebral gray matter density change in progressive supranuclear palsy using voxel based morphometry analysis and cerebral MR T1-weighted FLAIR imaging. *Journal of the Neurological Sciences*, *359*(1–2), 367–372. https://doi.org/10.1016/j.jns.2015.11.007

Wang, X., Zhang, J., Yuan, Y., Li, T., Zhang, L., Ding, J., Jiang, S., Li, J., Zhu, L., & Zhang, K. (2017). Cerebral metabolic change in Parkinson's disease patients with anxiety: A FDG-PET study. *Neuroscience Letters*, *653*, 202–207. https://doi.org/10.1016/j.neulet.2017.05.062

Whitwell, J. L., Duffy, J. R., Strand, E. A., Machulda, M. M., Senjem, M. L., Gunter, J. L., Kantarci, K., Eggers, S. D., Jack, C. R., & Josephs, K. A. (2013). Neuroimaging comparison of primary progressive apraxia of speech and progressive supranuclear palsy. *European Journal of Neurology*, *20*(4), 629–637. https://doi.org/10.1111/ene.12004

Xuan, M., Guan, X., Huang, P., Shen, Z., Gu, Q., Yu, X., Xu, X., Luo, W., & Zhang, M. (2019). Different patterns of gray matter density in early- and middle-late-onset Parkinson's disease: a voxel-based morphometry study. *Brain Imaging and Behavior*, *13*(1), 172–179. https://doi.org/10.1007/s11682-017-9745-4
